# Basal body positioning and anchoring in the multiciliated cell *Paramecium tetraurelia*: roles of OFD1 and VFL3

**DOI:** 10.1186/s13630-017-0050-z

**Published:** 2017-03-30

**Authors:** Hakim Bengueddach, Michel Lemullois, Anne Aubusson-Fleury, France Koll

**Affiliations:** Institute for Integrative Biology of the Cell (I2BC), CEA, CNRS, Université Paris Sud, Université Paris-Saclay, 1 Avenue de la Terrasse, 91198 Gif sur Yvette, France

**Keywords:** Basal body, OFD1-VFL3/CCDC61, Anchoring, Rotational asymmetry

## Abstract

**Background:**

The development of a ciliary axoneme requires the correct docking of the basal body at cytoplasmic vesicles or plasma membrane. In the multiciliated cell *Paramecium,* three conserved proteins, FOR20, Centrin 2, and Centrin 3 participate in this process, FOR20 and Centrin 2 being involved in the assembly of the transition zone. We investigated the function of two other evolutionary conserved proteins, OFD1 and VFL3, likely involved in this process.

**Results:**

In *Paramecium tetraurelia*, a single gene encodes OFD1, while four genes encode four isoforms of VFL3, grouped into two families, VFL3-A and VFL3-B. Depletion of OFD1 and the sole VFL3-A family impairs basal body docking. Loss of OFD1 yields a defective assembly of the basal body distal part. Like FOR20, OFD1 is recruited early during basal body assembly and localizes at the transition zone between axoneme and membrane at the level of the microtubule doublets. While the recruitment of OFD1 and Centrin 2 proceed independently, the localizations of OFD1 and FOR20 at the basal body are interdependent. In contrast, in VFL3-A depleted cells, the unanchored basal bodies harbor a fully organized distal part but display an abnormal distribution of their associated rootlets which mark their rotational asymmetry. VFL3-A, which is required for the recruitment of Centrin 3, is transiently present near the basal bodies at an early step of their duplication. VFL3-A localizes at the junction between the striated rootlet and the basal body.

**Conclusion:**

Our results demonstrate the conserved role of OFD1 in the anchoring mechanisms of motile cilia and establish its relations with FOR20 and Centrin 2. They support the hypothesis of its association with microtubule doublets. They suggest that the primary defect of VFL3 depletion is a loss of the rotational asymmetry of the basal body which specifies the sites of assembly of the appendages which guide the movement of basal bodies toward the cell surface. The localization of VFL3 outside of the basal body suggests that extrinsic factors could control this asymmetry.

**Electronic supplementary material:**

The online version of this article (doi:10.1186/s13630-017-0050-z) contains supplementary material, which is available to authorized users.

## Background

Generally absent in land plants or higher fungi, one or several cilia involved in motility, feeding, sensation, and sexual process, localize at the cell surface of numerous protists. This diversity is also observed in metazoa in which the number of cilia protruding from the surface depends on the cellular type: either a solitary cilium, also called primary cilium or several, often hundred cilia, can develop at the surface of specialized differentiated cells. Cilia are anchored at the cell surface by the basal body. In the case of the primary cilium, it is a derivative of the mother centriole, one of the two centrioles which organize the centrosome and contains two specific structures, the distal and sub-distal appendages.

Depending on the cellular type, the development of the primary cilium is initiated in the cytoplasm after docking of the mother centriole, via its distal appendages to Golgi derived vesicles or, directly in the external cellular space, after docking at the plasma membrane [1, 2]. This second pathway is also observed during the formation of motile cilia in multiciliated cells which occurs after multiplication of the centrioles/basal bodies deep in the cytoplasm and their migration towards the apical membrane. In multiciliated cells including epithelial cells of the human oviduct [3], of the epidermal Xenopus embryo [4] and of the mouse trachea [5], observations of basal bodies whose distal parts were surrounded by large or small vesicles suggest that in some cases, a basal body/vesicle interaction could also precede the docking of the basal body at the cell surface. The key role of the distal appendages in the anchoring process is supported by several results demonstrating that the depletion of proteins involved in their assembly impairs the docking [6].

In the multiciliated organism *Paramecium,* basal body anchoring at the cell surface does not involve vesicular intermediates [7]. The ciliogenesis is initiated by the development, from an already anchored basal body, of a new basal body which directly docks at the surface. Ciliated and non-ciliated basal bodies are observed indicating that axoneme extension is not necessarily associated with the basal body docking event [8]. Three typical plates organize the transition zone of the ciliated basal body, which also displays transition fibers, a ciliary necklace and Y links [9]. These three plates appear more closely apposed at the distal end of non-ciliated basal body and form the pro-transition zone [10]. These structures cap the tip of the basal body before its docking at the cell surface [7].

Three appendages protrude asymmetrically from the *Paramecium* basal bodies, one striated rootlet and two microtubular ribbons. They are thought to act as a scaffold for defining the site of new basal body assembly and to maintain the organization of the basal body row at the cell surface [11, 12]. A transient appendage of the mother basal body, the anterior left filament (ALF) also guides the movement of the daughter basal body toward the cell surface [13].

We have previously shown that Centrin 2, Centrin 3, and FOR20 are involved in the positioning and anchoring of basal bodies at the surface [8, 14]. Centrin 2 and FOR20 localize, respectively, in the basal body lumen and at the distal part of anchored basal body. The assembly of the structural elements of the pro-transition zone is impaired upon Centrin 2 and FOR20 depletion. Thus, in *Paramecium* as in metazoan cells, there is a correlation between defects in the structure of the basal body tip and in the anchoring process. In contrast, the depletion of Centrin 3 which is mainly located anteriorly to the proximal part of the centriolar cylinder [14] does not lead to defects in this assembly. The fully assembled new basal bodies are disoriented with respect to the mother, suggesting that the initial defect resides in the movement of the new basal body towards its docking site. This is supported by the fact that inactivation of Centrin 3 inhibits the formation of the ALF which may contribute to tilt up the basal body toward the cell surface [13].

In order to go further in the anchoring process in *Paramecium,* we undertook a functional analysis of homologs of conserved protein likely involved in this mechanism such as OFD1 and VFL3. OFD1, a protein whose mutations induce ciliopathies such as oro-facial-digital syndrome type 1 [15] is well known to be involved in the basal body anchoring process during the formation of the primary cilium. Located at the distal end of the mother centriole in mammalian cells [16], it is essential for the formation of the distal appendages which anchor the ciliary membrane vesicles before axoneme extension. Like FOR20, OFD1 possesses a conserved TOF/LisH motif [17] required for centrosomal localization [18]. OFD1 was recently shown to form a ternary complex with FOR20 and OFIP at the centriolar satellites of mammalian cells [19]. However the function of OFD1 in the assembly of the motile cilium remains relatively unstudied. *VFL3* was thoroughly studied in *Chlamydomonas.* Its mutation which affects basal body positioning at the cell surface is correlated with the lack of appendages [20]. Its ortholog in metazoa, CCDC61, is associated with the centrosome in human cells [21, 22]. We present here the analysis of OFD1 and VFL3 in *Paramecium*.

## Methods

### Strains and culture conditions

Stock d4-2 of *Paramecium tetraurelia*, the wild-type reference strain, was used in RNAi experiments. The mutant nd7-1 [23] which carries a recessive monogenic mutation preventing trichocyst discharge, a dispensable function under laboratory conditions, was used for transformations. Cells were grown at 27 °C in a wheat grass powder infusion, (Bio Herbe de Blé L’arbre de vie, Luçay Le Male, France) bacterized with *Klebsiella pneumoniae* and supplemented with 0.8 µg/ml β-sitosterol according to standard procedures [24].

### Gene cloning

Constitutive expression of the Myc-tagged *VFL3*-*1* and -*VFL3*-*3* genes: the tag was added at the 5′ end of each gene by PCR amplification, using 5′-end specific primers in which the Myc coding sequence was added. After restriction digests, the fragments were cloned into the *Spe*I restriction site of the pPXV vector, the recombinant genes being under the control of the *Paramecium* calmodulin constitutive regulators.

Expression of the *GFP*-*VFL3*-*3* and *GFP*-*OFD1* under the control of their own regulators: the putative promoter of each gene was first amplified by PCR and cloned at the 3′ end of the GFP-coding fragment inserted into the pZZ-GFP vector (a derivative of the pPXV-GFP vector kindly provided by Jean Cohen) using the *BamH*1 and *Spe*I restriction sites. Then, each gene, followed by its putative 3′ UTR, was amplified by PCR and cloned at the 3′ end of the GFP sequence, between the *Xho*1 and *Xma*I sites.

Constitutive expression of *GFP*-*OFD1*: the *OFD1* gene was amplified by PCR from genomic DNA and cloned downstream the GFP sequence into the *Kpn*I site of the pPXV-GFP plasmid which contains the constitutive regulators of the calmodulin gene. Three glycine codons were added between the *GFP* and the *OFD1* sequences.

Gene silencing: all RNAi plasmids are derivatives of the vector L4440 [25] and carry fragments of the target genes inserted between two convergent T7 promoters. Silencing of the *VFL3*-*A* subfamily genes was performed using a vector containing a fragment of the *VFL3*-*1* gene (from base 20 to 637) linked to a fragment of the *VFL3*-*2* gene (from base 861 to 1375). The two fragments were amplified by PCR and cloned successively into the vector. The RNAi of the *VFL3*-*B* subfamily was performed using a vector containing a *VFL3*-*3* fragment extending from base 328 to 701 and linked to a part of the *VFL3*-*4* gene extending from base 1124 to 1725 (see Additional file 1: Figure S1). Finally, for *OFD1* silencing, a fragment extending from base 254 to 1310 was used. Amplifications were performed with high fidelity Phusion DNA polymerase using standard procedures. After cloning, the genes were entirely sequenced to ensure that no error was introduced during the amplification. Possible RNAi-off target effects were analyzed for each construct using the RNAi-off target tool available at ParameciumDB [26].

### RNA extraction and Northern blot analysis

Total RNA was extracted using TRIzol as previously described [27]. For Northern blots, 20 μg of total RNA were denatured and loaded on 1% agarose gels. Electrophoresis in 1× Tris–borate–EDTA transferred to Hybond N+ membranes (GE Healthcare), and hybridization with 32P-labeled DNA probes were performed as previously described [28]. *VFL3*-*1* probe: sequence of the *VFL3*-*1* gene from base 861 to 1375. *VFL3*-*2* probe: sequence of *VFL3*-*2* from base 20 to 637. *VFL3*-*3* probe: sequence of *VFL3*-*3* from base 1354 to 1725. *VFL3*-*4* probe: sequence of *VFL3*-*4* from base 330 to 831. The sequences of these probes are specific for each gene and should not cross-react with the double strand RNA produced by the RNAi vector. Radioactive signals were quantified using ImageQuant.

### Paramecium transformation

nd7-1 mutant cells unable to discharge their trichocysts were transformed by microinjection into their macronucleus of filtered and concentrated plasmid DNA containing a mixture of the plasmids of interest (5 µg/µl) digested by *Sfi*1 and of plasmid DNA directing the expression of the *ND7* wild-type gene [23]. Transformants were first screened for their ability to discharge their trichocysts and if so, further analyzed. Microinjection was made under an inverted Nikon phase-contrast microscope, using a Narishige micromanipulation device and an Eppendorf air pressure microinjector.

### Gene silencing

Gene silencing was performed by the feeding method as previously described [29]. Stationary wild-type cells or growing transformant cells were fed individually with the appropriate double-stranded RNA-expressing HT115 bacteria. They gave rise to independent cell lines containing a population of cells which presented a homogenous phenotype. Growing cells were transferred daily into fresh feeding medium as needed. Control cells were fed with bacteria transformed by the L4440 plasmid or a plasmid containing the *ND7* gene involved the exocytosis [23]. Generally, at least three independent experiments were conducted, and routinely, 6–9 clones obtained from each independent experiment were analyzed.

### Immunolabeling

Each immunolabeling was carried out on samples of 20–50 cells as previously described [30]. Briefly, paramecia were permeabilized 5 min in 1% TritonX100 in PHEM buffer (PIPES 60 mM, HEPES 25 mM, EGTA 10 mM, MgCl_2_ 2 mM pH 6.9) fixed in 2% paraformaldehyde in PHEM buffer for 10 min, and rinsed 2 × 15 min in TBST/BSA (Tris 10 mM, NaCl 0.15 M, Tween20, 3% BSA, EGTA 10 mM MgCl_2_ 2 mM). All subsequent steps were performed in this same buffer. Cells were incubated in the primary antibody for 20 min, rinsed twice and incubated in the secondary antibody for 25 min, rinsed in TBST/BSA supplemented with Hoescht 2 mg/ml and then in TBST/BSA without Hoechst.

#### Antibodies

The monoclonal anti-tubulin antibody 1D5 [31] at a dilution 1:100; a polyclonal affinity purified PolyE anti-tubulin [32] at 1:7000 dilution; the monoclonal CTS32 directed against epiplasmins diluted 1:20 to decorate the epiplasm [33], the monoclonal anti-Myc antibody 9E10 (Sigma) at a dilution 1:1000, the polyclonal anti-ciliary rootlet serum (KD2) [34] at a dilution 1:800, the monoclonal anti-tubulin acetylated TEU318 [35] culture supernatant at a dilution 1:10, and secondary antibodies labeled with Alexa Fluor 488 or 568 from Invitrogen-Molecular Probes (Eugene, OR), or Cy5 (Life Technologies) at a dilution 1:250–1:500.

### Fluorescence microscopy

Cells were observed with a Zeiss Axioskop 2 plus equipped with epifluorescence. Images were acquired with a CoolSnap camera coupled with Metavue. Confocal acquisitions were made with a Leica SP8 equipped a UV diode (line 405), and three laser diodes (lines 488, 552 and 635) for excitation and two PMT detectors. Images stacks were processed with imageJ and Photoshop. STED imaging was performed using a Leica TCS SP8 STED 3X (Leica Microsystems CMS GmbH, Mannheim, Germany). The system was equipped with a WLL ranging from 470 to 670 nm for excitation and with 3D STED lasers at 592, 660, and 775 nm. A 100× 1,4 Oil STED white objective was used to acquire the images. GFP, AF568, and Cy5 were excited at 488, 561, and 643 nm, respectively. Detection ranges were 500–550, 575–625, and 660–700 nm, respectively. A pixel size of 25 nm was used. For deconvolution, SVI Huygens was used.

### Countings of basal bodies

Countings of basal body doublets were performed from projections of images acquired at the dorsal side of paramecium cells labeled by the 1D5 antibody. Automatic countings were performed by using the ImageJ software and were refined manually. Seven wild-type cells and eleven *VFL3*-*A* inactivated cells obtained from three independent RNAi knockdown experiments were used. The percentage of basal body containing abnormal striated rootlets was calculated from manual counting on ten cells obtained from three independent RNAi knockdown experiments.

### Electron microscopy

For ultrastructural observations, the depleted and control cells were fixed in 1% (v/v) glutaraldehyde and 1% OsO4 (v/v) in 0.05 M cacodylate buffer, pH 7.4 for 30 min. After rinsing and dehydration in ethanol and propylene oxide series, they were embedded in Epon resin. For pre-embedding immunolocalization, the immunostaining process was carried out as described for immunofluorescence using a gold-coupled instead of fluorochrome-coupled secondary antibody. Then the cells were rinsed, fixed and embedded as previously described [8]. For post-embedding immunolocalization, cells were fixed in 3% paraformaldehyde, 0.15% glutaraldehyde, in 0.05 M cacodylate buffer, pH 7.4 at 4 °C for 1 h. After washes and dehydration in ethanol, they were embedded in LR White (London Resin). Thin sections were collected on nickel grids and treated with 0.1 M NH_4_Cl in 0.1 M PBS and then saturated with 3% BSA and 0.1 M glycine in PBS. Sections were incubated with anti-GFP polyclonal antibody at room temperature for 45 mn. After several washes in PBS, a gold-labeled anti-rabbit IgG (GAR G10, Aurion) diluted 1/50 was applied for 30 mn. The grids were rinsed in PBS and distilled water, and finally stained with uranyl acetate. All ultrathin sections were contrasted with uranyl acetate and lead citrate. The sections were examined with Jeol 1400 (120 kV) or Philips CM10 transmission electron microscopes.

## Results

### Identification of the VFL3 and OFD1 genes in *Paramecium tetraurelia*

While a single *VFL3 * gene is present in *Chlamydomonas*, four *VFL3* genes (*VFL3*-*1*, *VFL3*-*2 VFL3*-*3*, and *VFL3*-*4*) are encoded in the *P. tetraurelia* genome which has undergone at least three successive whole-genome duplications (WGD) during the evolution [27]. Phylogenetic analysis indicates that the four *P. tetraurelia* genes can be grouped into two families (*VFL3*-*A* and *VFL3*-*B*), the VFL3-A proteins being more closely related to the *Chlamydomonas* one (Additional file 2: Figure S2). Only one gene of each family is encoded in the *Paramecium caudatum* genome, a closely related species which shares only the most ancient of the three WGDs with *P. tetraurelia* [36]. Only one *OFD1* ortholog is encoded in the genome. The alignment of the conserved N-terminal part of this protein with its orthologs is presented in Additional file 3: Figure S3.

### Role of OFD1 in the basal body anchoring process and distal part assembly

We tested the involvement of *OFD1* in basal body anchoring by RNAi knockdown experiments using the feeding method [29]. To silence the unique *OFD1* gene, we performed inactivation by bacteria producing dsRNA covering 56% of the gene (1055/1887 nt). The efficiency of the vector to inactivate its target was tested by controlling for the effective depletion of the GFP-tagged protein (see Additional file 4: Figure S4**)**.

In interphase cells, the basal bodies are embedded in cortical units which contain either one or two basal bodies, the distribution of these units being specific of the cell areas. During cell division, neoformed basal bodies are inserted in these units anterior to their parental basal bodies. Depending on the cell territories, several rounds of duplication can occur, generating several new basal bodies from a preexisting one. This is associated with the formation of the new cortical units in the daughter cells [37]. An additional wave of duplication occurs in a subset of cortical units to generate units with basal body pairs. After division, parental and newly assembled basal bodies alternate within the rows and the daughter cells display a global pattern of cortical units identical to that of their mother [11].

**Fig. 1:**
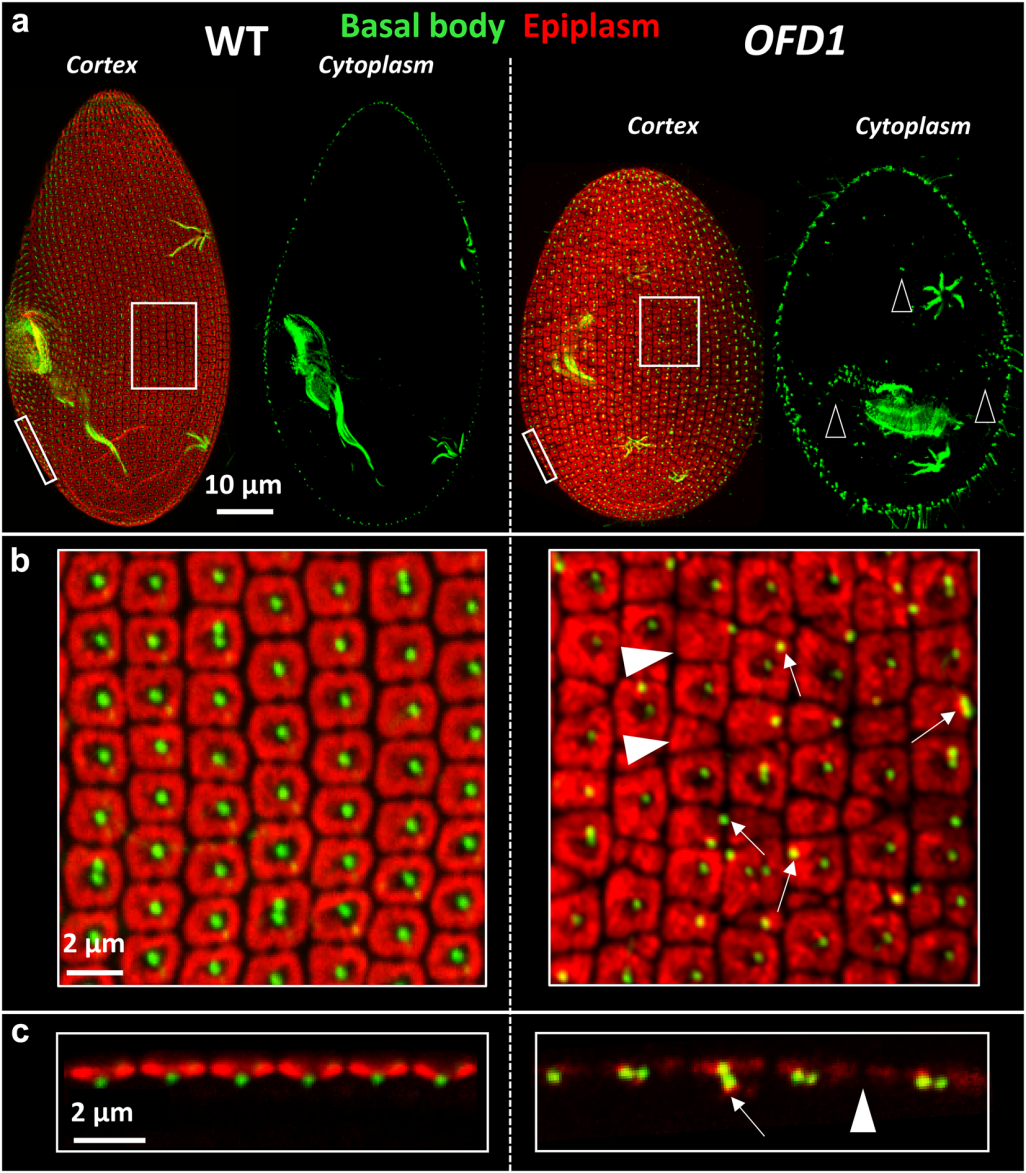
OFD1 depletion alters basal body positioning. **a** Projections of optical sections through the dorsal surface (cortex) and the cytoplasm of cells labeled with 1D5 (*green*), a marker of basal bodies and the anti-epiplasm antibody, a marker of the cortical units (*red*). *OFD1* cell observed after one division upon OFD1 depletion. The depleted cell is smaller than the control. Numerous internal basal bodies are observed (*empty arrowheads*). **b** Magnification of **a**. The basal bodies (*green*) appear in the center of the units (*red*) in the wild-type cell. Basal bodies lying between units (*arrows*) and units devoid of basal bodies (*filled arrowheads*) are observed in the OFD1 depleted cell. **c** Optical transverse sections (*top* outside, *bottom* inside the cell) performed across the cell surface. In the control cell, the basal bodies are located at the center of the epiplasmic units (*red*). In the OFD1 depleted cell, basal bodies lying under the cell surface are observed (*white arrows*). Small epiplasmic units and epiplasmic units devoid of basal bodies are also observed (*filled arrowhead*)

**Fig. 2:**
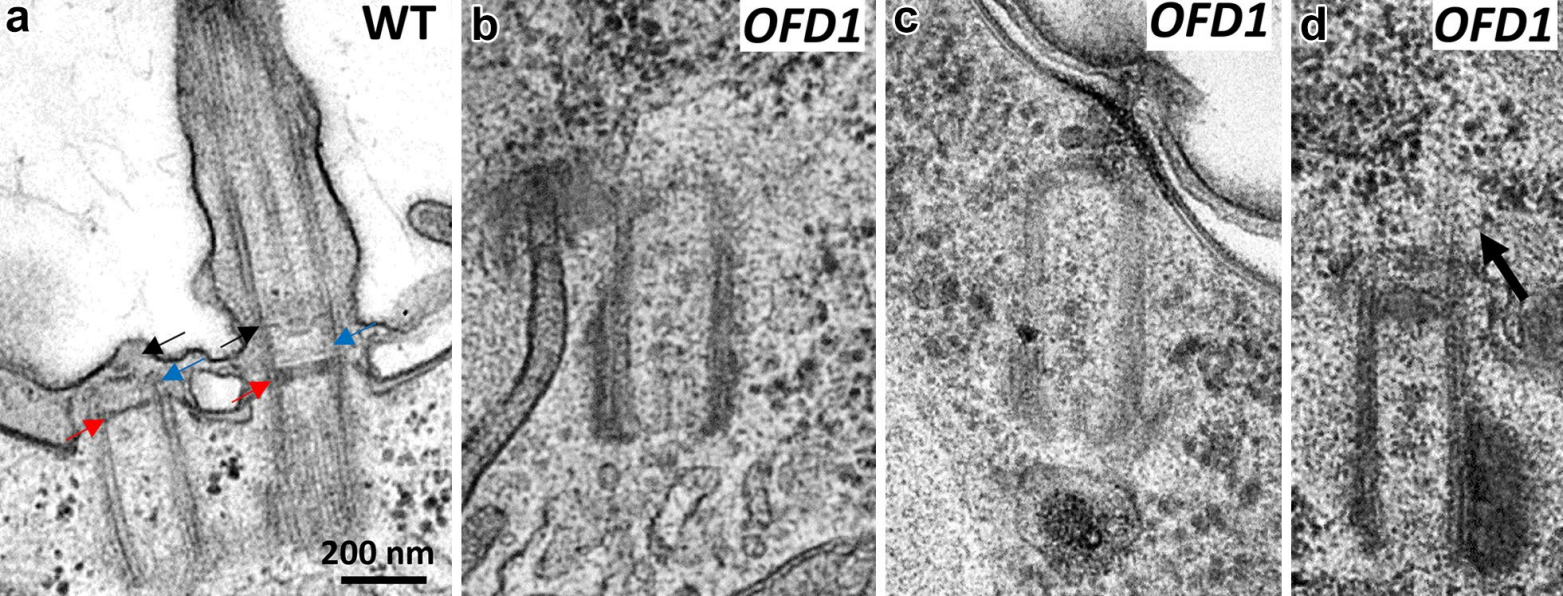
Anomalies of basal body ultrastructure induced by OFD1 depletion. Longitudinal sections of non-ciliated and ciliated basal bodies in WT cell (**a**) and OFD1 depleted cell (**b**–**d**). The three plates present in the pro-transition zone and the transition zone (**a**) are indicated by *arrows*. *Red arrows* terminal plate, *blue arrows* intermediate plate, *black arrows* axosomal plate. Undocked (**b**) or partially docked (**c**) basal bodies in OFD1 depleted showing defective distal structures. **d** An internal basal body prolongated by an abnormal transition zone and microtubules (*black arrow*)

*OFD1* knockdown induced a cellular growth defect and cell size reduction after 1 division and cellular death after 4/5 divisions in all the analyzed clones (*n* > 24) (see “Methods”). To analyze the effects of the depletion on the basal bodies, inactivated cells were labeled by the 1D5 antibody which decorates the basal bodies and an anti-epiplasm antibody specific of the subcortical units. Figure 1 shows the defects induced by OFD1 depletion after one division upon silencing. Many basal bodies are detected in the cytoplasm of OFD1 depleted cells (Fig. 1a right panel). Whereas in the control cells, the basal bodies are located in the middle of the cortical units, in OFD1 depleted cells, basal bodies located between the units or lying under the cell surface are observed (Fig. 1b, c). At the ultrastructural level, whether undocked or partially docked at the membrane, basal bodies (*n* = 23) show defective distal structures (Fig. 2b, c). The three plates of the transition zone are often absent (65%; *n* = 15) or partially assembled (35%; *n* = 8). Rare intracytoplasmic undocked basal bodies prolongated by microtubules are also detected which could result from a premature extension of the basal bodies (Fig. 2d).

### OFD1 is recruited early during basal body duplication and localizes at the transition zone proximal part after anchoring

**Fig. 3:**
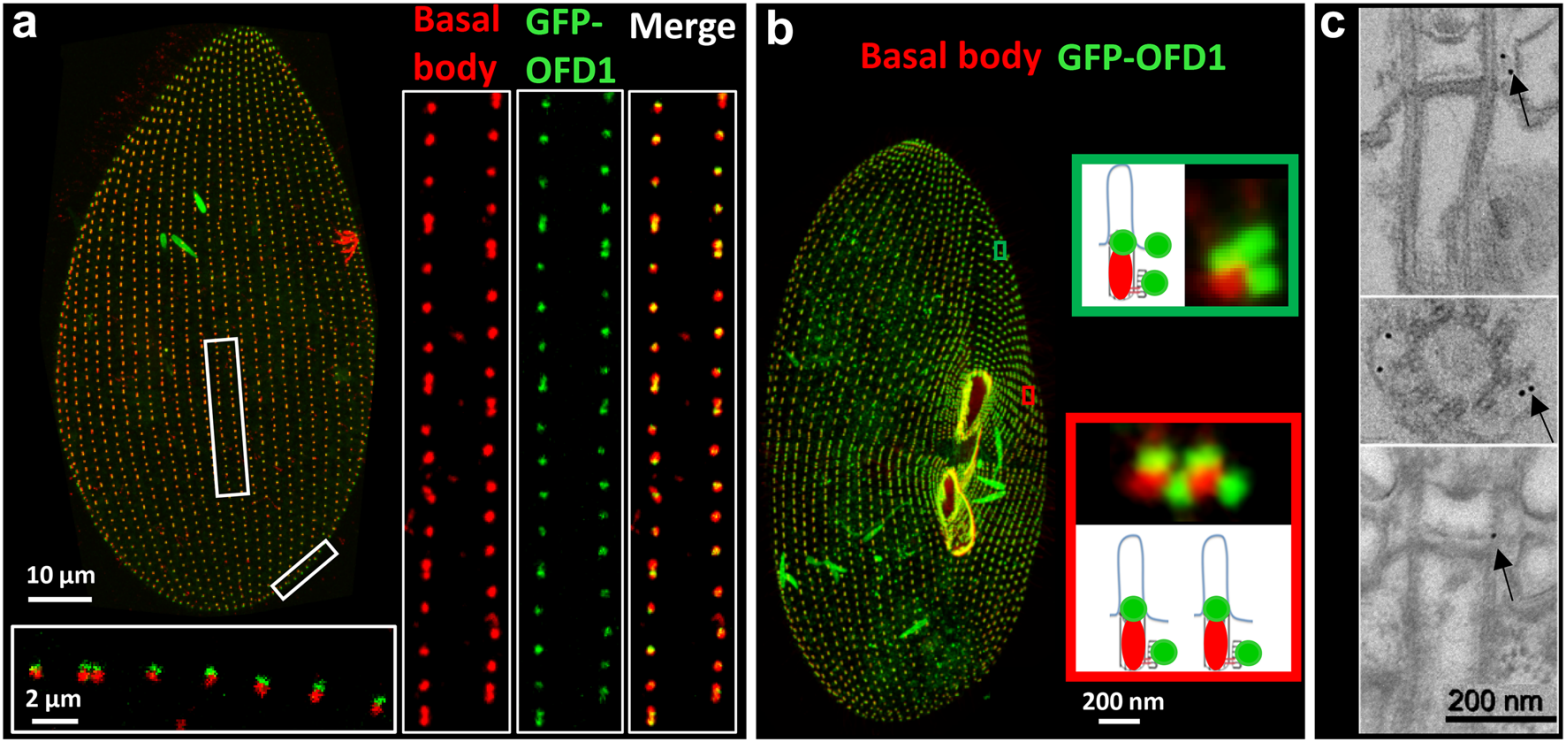
Localization of GFP-OFD1. **a**, **b** Immunofluorescence. Projection of confocal section through the dorsal (**a**) or the ventral (**b**) surface of transformants expressing GFP-OFD1 stained by 1D5. **a** An interphase cell. *Right insets* the GFP-OFD1 signal (*green*) overlaps the 1D5 labeling (*red*) on all basal bodies. *Bottom inset* an optical transverse section (*top* outside, *bottom* inside the cell) performed across the cell surface shows the GFP signal at the distal part of all basal bodies. **b** A dividing cell. *Red inset* an optical section through the cell surface at the level of duplicating basal bodies. In addition to the distal GFP labeling (*green*) at the distal part of the parental basal bodies (*red*), the GFP signals, not labeled by 1D5, localize at the site where new basal bodies are expected to assemble. *Green inset* an optical section at the level of the “invariant field.” A dual GFP labeling is detected near the posterior basal body of the doublet when the new basal body assembles: one corresponding to the nascent basal body and the other one corresponding to the anchoring site of the disassembled one. **c** Immunoelectron microscopy. Localization of the GFP tag after pre-embedding (*upper panel*) and post-embedding (*lower panel*) immunolabeling. On longitudinal sections, the labeling is located just above the terminal plate near the intermediate plate (*arrows*). The gold particles are always localized to the outer side of the axoneme at the level of the microtubular doublets

To ascertain the localization of OFD1, we expressed a GFP-tagged protein either under the control of its own regulators or under the control of the constitutive calmodulin gene-regulatory elements. After transformation, we observed the signal on transformants displaying a wild-type growth rate and phenotype. Whatever the vectors used for driving the expression of the tagged protein, the fluorescence localized at the distal end of all basal bodies as demonstrated by labeling with 1D5 (Fig. 3a).

In *Paramecium,* the basal body duplication process starts at the equatorial zone and propagates towards the cell poles according to a precise spatio-temporal pattern. Consequently, in a dividing cell, several types of cortical units containing basal bodies at different stages of the duplication process coexist: unduplicated, duplicating and fully assembled. Because several rounds of duplication occur during cell division, one basal body can give rise to several new ones, yielding clusters of four to six basal bodies. Observations of dividing transformants (Fig. 3b) allowed us to confirm that, as already described for FOR20 [8], OFD1 is recruited early during basal body biogenesis before its anchoring at the cell surface. Indeed GFP fluorescent dots, not yet labeled by the anti-tubulin 1D5, are observed close to the preexisting basal bodies, at the position corresponding to the assembly site of the new basal body (Fig. 3b red inset). In the anterior part of the cells, a subset of basal body doublets does not follow a classical duplication pattern. In this region, the anterior basal body of each pair disassembles, its disappearance being followed by the assembly of a new basal body which replaces the disassembled one [38]. In contrast to what is observed in the other parts of the cell (Fig. 3b: green inset vs red inset), a dual GFP labeling is detected near the posterior basal body of the pair when the new basal body assembles: one corresponding to the nascent basal body and the other one corresponding to the anchoring site of the disassembled one. This indicates that OFD1 is stably incorporated at the cell surface as previously shown for FOR20.

We ascertained the localization of OFD1 at the ultrastructural level (Fig. 3c). No morphological basal body defect was observed in transformants expressing GFP-tagged OFD1. Gold particles were detected in about 50% (*n* = 24) and 30% (*n* = 14) of basal body longitudinal sections observed after pre- and post-embedding processing, respectively. 87% (pre-embedding) and 95% (post-embedding) of the gold particles were located in the basal body distal part corresponding to the transition zone. Most of these particles localize between the axoneme and the membrane above the terminal plate which marks the limit between the basal body microtubule triplets and the axonemal doublets. This localization is similar to that observed for FOR20.

### Relationships between OFD1–FOR20–Centrin 2

We then investigated the relationships between OFD1 and both FOR20 and Centrin 2 which are also required for assembly of the basal body distal part. We followed the fate of the fluorescence in GFP-OFD1 transformants after knockdown of FOR20 and Centrin 2 and carried reciprocal experiments in which OFD1 was silenced in transformants expressing GFP-FOR20 or GFP-Centrin 2 (Additional file 5: Figure S5). After two or three divisions under RNAi conditions, all basal bodies retained fluorescence in the GFP-OFD1 transformants silenced for Centrin 2 as well as in GFP-Centrin 2 transformants inactivated for OFD1. This indicates that there is no relationship between OFD1 and Centrin 2 for their recruitment at the basal bodies. In contrast, the GFP signal decreases in transformants expressing GFP-OFD1 silenced for FOR20 and in GFP-FOR20 transformants depleted of OFD1. This reveals an interdependence between OFD1 and FOR20 for their localization/stability at the basal bodies.

### The *VFL3*-*A* gene family is involved in basal body anchoring

To ascertain the function of the *VFL3*-*A* and *VFL3*-*B* genes, two different RNAi vectors targeting specifically and simultaneously both genes of each family were used. As observed for OFD1, the knockdown of the *VFL3*-*A* genes induces a growth defect and size reduction after 1 division and cellular death after 4/5 divisions on all the analyzed clones (*n* > 30) see “Methods”. In contrast, inactivation of the *VFL3*-*B* genes does not produce any detectable phenotype. The efficiency and specificity of each vector to silence its targets was confirmed by controlling the effective messenger depletion of the four *VFL3* genes in response to the inactivation (see Additional file 6: Figure S6). A very weak signal was detected with the probe corresponding to the *VFL3*-*4* gene (VFL3-B family) in the control as well as in the silenced cells, indicating that the gene is probably expressed at a very low level. The lack of complementation of the *VFL3*-*A* genes by the expression of the *VFL3*-*3* isoform (VFL3-B family) indicates that the two *VFL3*-*A* and *VFL3*-*B* gene subfamilies have different functions. The 63% reduction of the expression of the *VFL3*-*3* gene does not seem sufficient to induce a cellular phenotype.

**Fig. 4:**
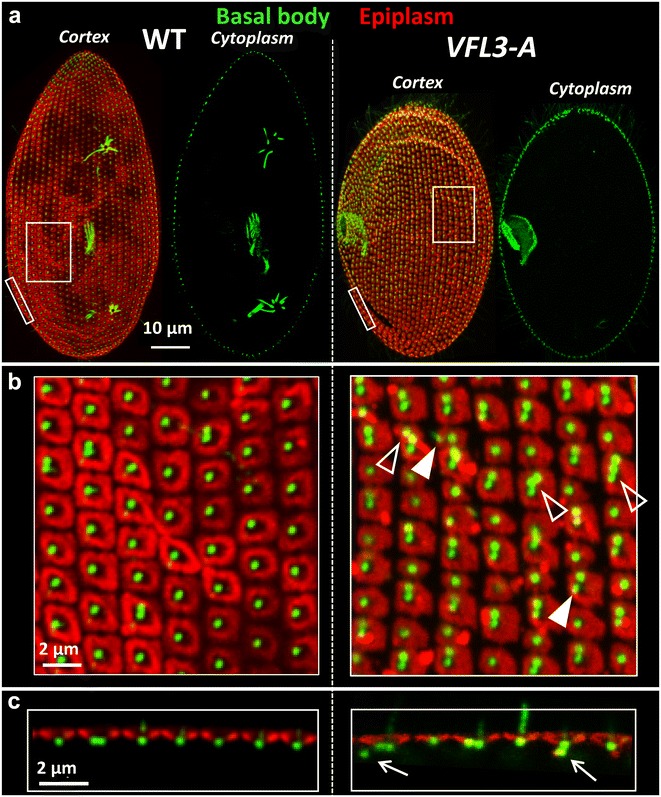
VFL3-A depletion alters basal body positioning. **a** Projections of confocal sections through the dorsal surface (cortex) and the cytoplasm of cells labeled with 1D5 (*green*), a marker of basal bodies and the anti-epiplasm antibody (*red*). *VFL3*-*A* cell observed after one division upon VFL3-A depletion. The depleted cell is smaller than the control. **b** Magnification of **a**. Singlets and some doublets appear in the center of the units in the wild-type cells. Numerous basal bodies doublets and some triplets (*empty arrowheads*) are observed in VFL3-A depleted cells. Some basal bodies are mispositioned (*filled arrowhead*). **c** Optical transverse sections (*top* outside, *bottom* inside the cell) performed across the cell surface. In the VFL3-A depleted cell, basal bodies lying under the cell surface are observed (*arrow*)

**Fig. 5:**
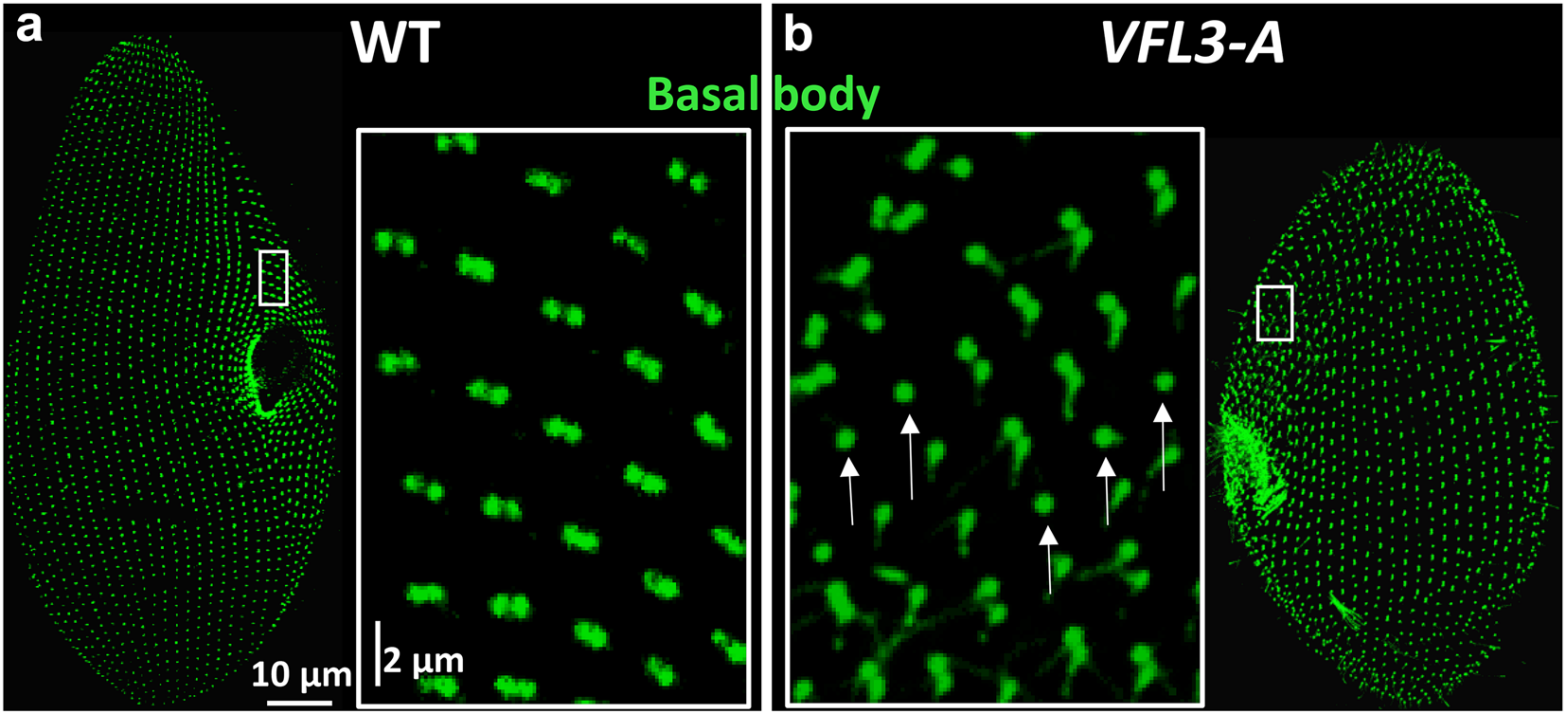
Basal body duplication is impaired upon *VFL3*-*A* depletion. **a** Projection of confocal section performed on **a** wild-type (WT) and **b** *VFL3*-*A* depleted cells labeled by the 1D5 antibody (*green*). The *insets* correspond to the “invariant fields,” a part of the cortex displaying only basal body doublets in wild-type cells **a**. Presence of singlets (*arrows*) in VFL3-A depleted cells **b** reveals anomalies in basal body duplication

The effect of VFL3-A depletion on the basal bodies was tested by immunostaining with the 1D5 antibody and the anti-epiplasm antibody. Contrary to what was observed in OFD1 depleted cells, in very rare cases basal bodies were present in the cytoplasm after the first division upon inactivation (Fig. 4a). However, basal bodies mispositioned on the cortex or lying beneath the cell surface (Fig. 4b, c) were observed. An excess of cortical units containing basal body pairs [32 ± 6% in inactivated cells (*n* = 11) vs 6 ± 1% in control cells (*n* = 7)] as well as clusters of three basal bodies within one cortical unit, never observed in wild-type interphase cells, were also associated with VFL3-A depletion. This excess can arise either from over-duplication during the assembly of the basal body pairs, or from a defect in the segregation of the new versus old basal bodies after their assembly. Examination of the so-called invariant field, a region where basal bodies are associated in pairs in all units, allowed us to eliminate the first hypothesis (Fig. 5). In this region, cortical units containing a single basal body were observed, indicating that the depletion of VFL3-A could affect in some way new basal body assembly.

### VFL3-A controls the correct assembly of the basal body proximal appendages

**Fig. 6:**
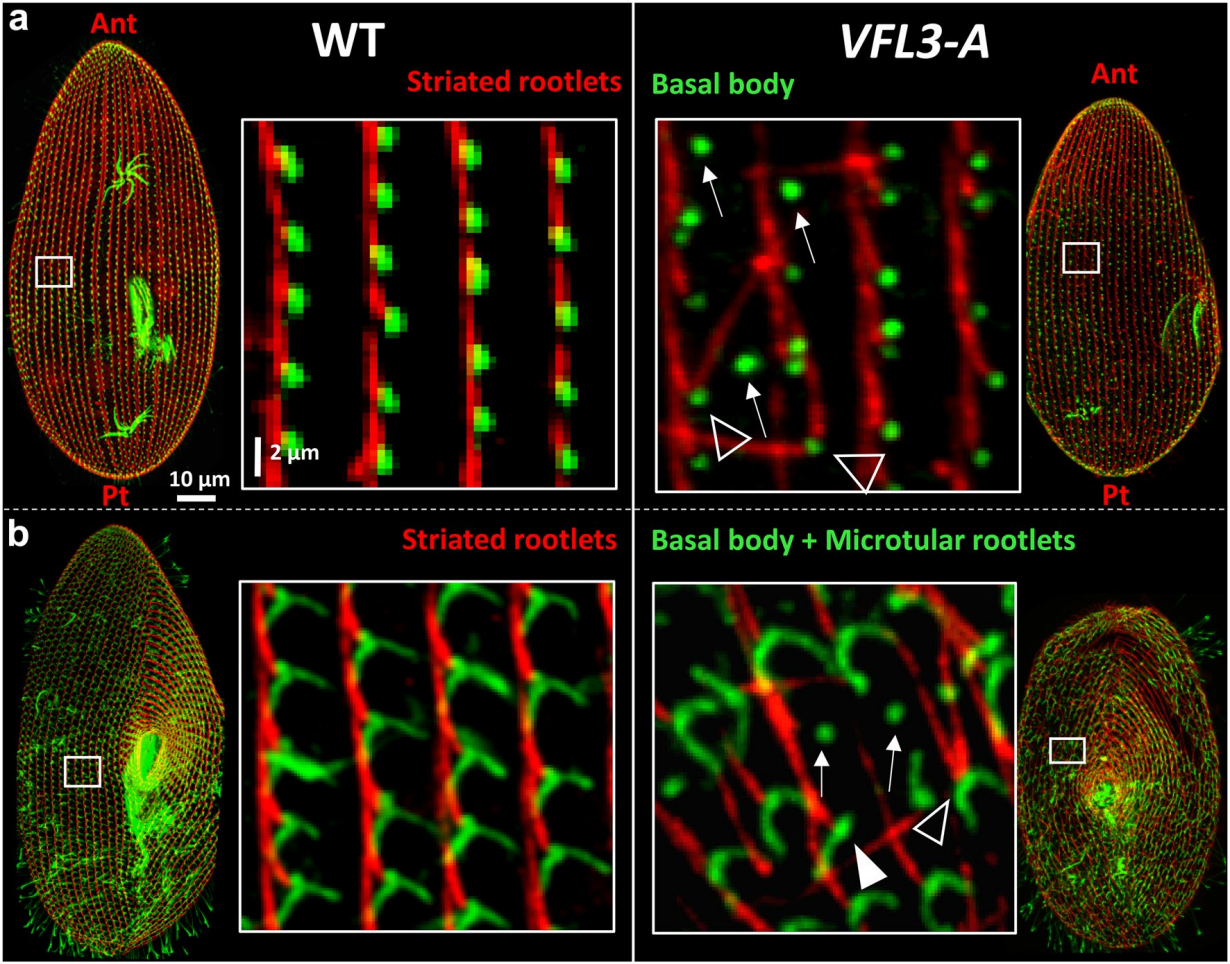
The assembly of basal body appendages is impaired in VFL3-A depleted cells. Projection of confocal sections performed on **a** wild-type (WT) and *VFL3*-*A* depleted cells double labeled with KD2 (*red*), a marker of the striated rootlets and 1D5, a marker of basal bodies (*green*). *WT* a unique striated rootlet marks the basal bodies. All striated rootlets point to the anterior part of the cell (Ant). *VFL3*-*A* the basal body mispositioning is associated with the misalignment of the striated rootlets. Some basal bodies are completely devoid of striated rootlet (*arrows*) and other assemble several striated rootlets (*arrowheads*). **b** Double staining of WT and VFL3-A depleted cells by KD2 (*red*) and TEU318, a marker of basal bodies and microtubular rootlets. WT in addition to the striated rootlet, two microtubular rootlets mark the basal bodies (*green*). *VFL3*-*A* basal bodies devoid of striated rootlet are generally not associated with microtubular rootlets (*arrows*). Some basal bodies are associated with a single microtubular rootlet (*white arrowhead*). *Pt* posterior part of the cell

In *Paramecium,* three appendages associated with the proximal part of the basal body organize the movements and separation of the basal bodies during the duplication process [39]: one striated rootlet and two microtubular ribbons. We thus analyzed the organization of these appendages in VFL3-A depleted cells. Double staining by KD2 (a marker of the striated rootlets) and 1D5 (a marker of basal bodies) or TEU318 (a marker of the microtubular ribbons) reveals abnormalities in the organization of these structures. The striated rootlets are disoriented relative to the cellular antero-posterior axis and pointed in all directions (Fig. 6a). More surprising is the presence of basal bodies devoid of striated rootlet (11.5 ± 2%) which generally also lack their microtubular appendages (Fig. 6b). In addition, we also observed basal bodies carrying more than one striated rootlet (3 ± 1.5%), a phenotype never observed in the wild-type strain. These alterations affect only the basal bodies assembled during the VFL3 depletion which represent approximatively 50% of the basal bodies present in the daughter cells after the inactivation. Thus, we can estimate that the number of basal bodies which form abnormal appendages during the VFL3-A depletion represents about 30% [(11.5% + 3%) × 2)] of the newly assembled basal bodies.

**Fig. 7:**
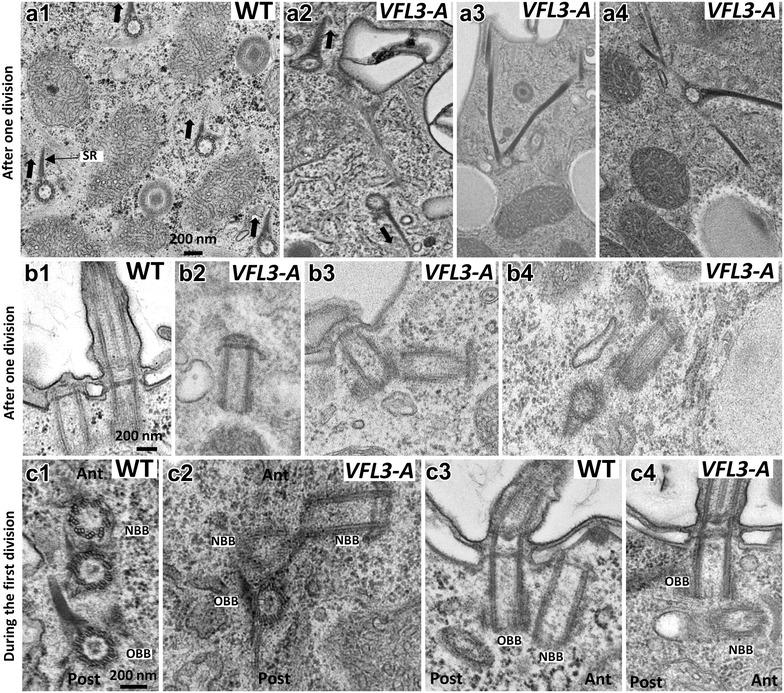
Ultrastructural anomalies of basal bodies induced by VFL3-A depletion. **a** Cross section of wild-type cell (*A1*): one striated rootlet (SR) is associated with basal body, all pointing in the same direction (*black arrows*). *VFL3*-*A* the striated rootlets are disoriented (*A2*) and two or three rootlets can develop from a basal body (*A3*, *A4*). **b** Longitudinal sections of basal bodies in wild-type cell (*B1*) and VFL3 depleted cells observed after one division upon inactivation (*B2*, *B3*, *B4*). *B1* Ultrastructure of non-ciliated and ciliated basal bodies in wild-type cell. *B2* 80% of the observed undocked basal bodies (*n* = 50) assemble normal pro-transition zone. *B3*, *B4* These sections reveal fully mature new basal bodies that did not tilt up to a position parallel to their mother. **c** Transversal and longitudinal sections of basal bodies during the duplication process in wild-type cell (*C1*, *C3*) and VFL3-A depleted cells (*C2*, *C4*). Cells were observed during the first division upon inactivation. In the wild-type cell, duplicating basal body are aligned (*C1*, *C3*). In VFL3 depleted cell, the new basal bodies are not separated from the parental one. The most anterior one do not tilt up to a position parallel to the old one. An abnormal filamentous structure links new and ancient basal body (*C2*). The longitudinal section reveals a new basal body that have developed in abnormal orientation (*C4*). *Ant* anterior part, *post*-posterior part

The ultrastructural analysis confirmed the basal body misorientation in *VFL3*-*A* inactivated cells and the defect in assembly of their striated rootlets. In the wild-type strain, a unique striated rootlet is associated with all basal bodies, all of them pointing to the same direction (Fig. 7A1). In addition to the defect in the orientation of these fibers (Fig. 7A2), more than one striated rootlet was found associated with some basal bodies in VFL3-A depleted cells (Fig. 7A3, A4). In most of the VFL3-A depleted cells observed after one division upon inactivation, the non-anchored basal bodies appeared fully assembled and harbored distal structures equivalent to the pro-transition zone present in the wild-type cells (Fig. 7B2). Observations on proliferating basal body units in cells undergoing their first division under VFL3-A depletion (Fig. 7C2, C4) revealed the presence of daughter basal bodies developing at inaccurate sites, with a mature length, but with abnormal orientation and unable to tilt up. Defect of spacing between old and new basal bodies as well as attachment of newly formed basal bodies to their parent by an abnormal filament were also observed as if the severing step was impaired.

### VFL3 localization

**Fig. 8:**
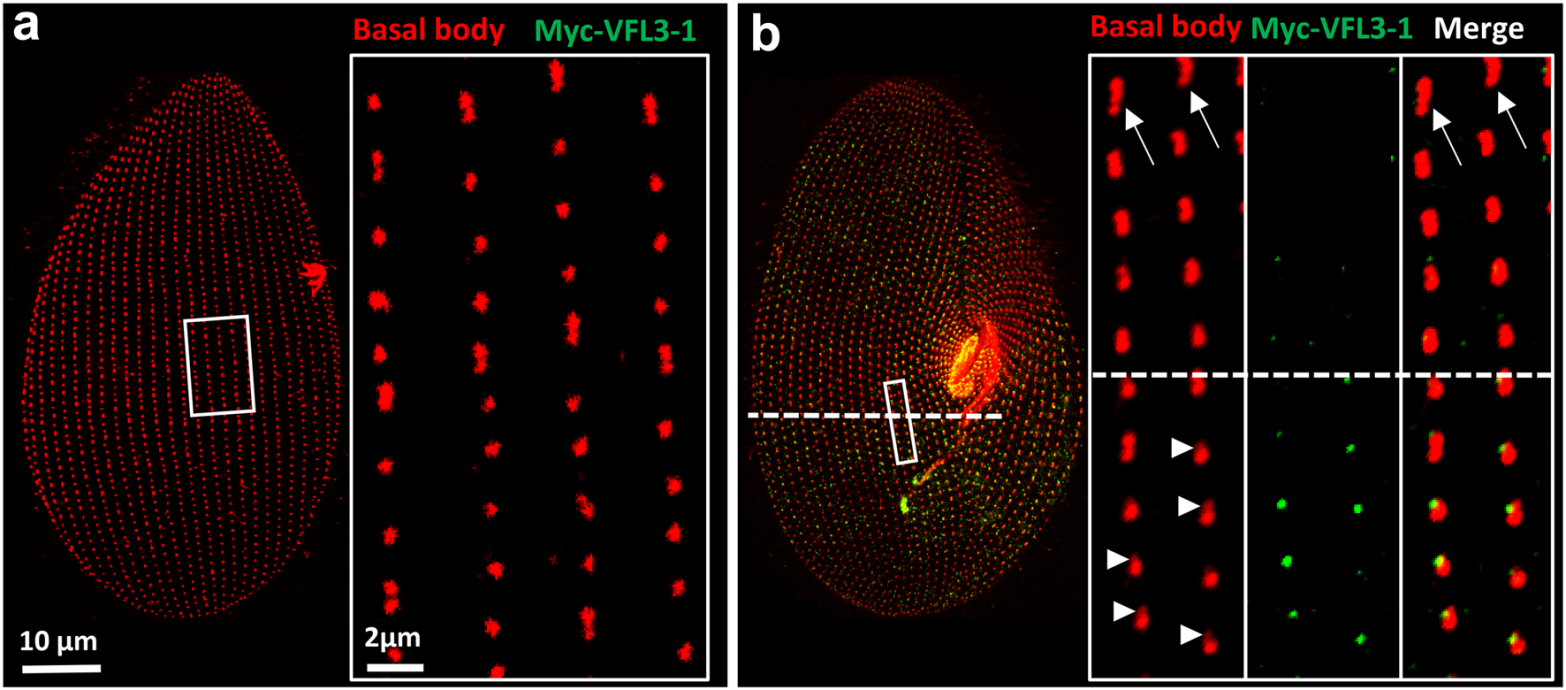
Myc-VFL3-1 is expressed during basal body duplication. Projections of confocal sections performed on transformant expressing the Myc-VFL3-1 protein stained by 1D5 (*red*) and anti-Myc antibody (*green*). **a** Interphase cell, **b** cell at an early stage of the division process. *Insets* show higher magnifications of the *white squares* on the corresponding cells. **a** In the interphase cell, only singlets and some doublets coexist in the surface (*right inset*). No Myc labeling is observed. **b** *Right inset*, *bottom* of the *dashed line* the basal bodies duplication is in progress as judged by the appearance of a 1D5 weak signal (*arrowhead*) anterior to the mother basal bodies. Myc labeling is observed. *Top* of the *dashed line* basal bodies are fully duplicated as judged by the presence of doublets and some triplets (*arrows*). No Myc labeling is observed

One gene of each *VFL3* family (*VFL3*-*1* and *VFL3*-*3*) was tagged by the GFP or Myc epitope. After transformation, we observed the signal on transformants which displayed a wild-type growth rate and phenotype. A very weak fluorescence associated with the basal bodies was observed at all stages of the cell cycle in transformants expressing GFP or Myc-tagged VFL3-3 (Additional file 7: Figure S7). In contrast, the Myc-tagged *VFL3*-*1* isoform is recruited to the cortex only during the division. No labeling is observed during the interphase (Fig. 8a), whereas double staining with the anti-Myc and 1D5 antibodies reveals a signal during the basal body duplication process (Fig. 8b). This anti-Myc staining was observed regardless of the regulatory sequences used for driving the expression of the fusion protein. Figure 8b illustrates the localization of Myc-VFL3-1 in a dividing cell at the onset of division. In the part of the cell where basal body duplication is in progress (below the dashed line), as judged by a weaker 1D5 labeling of the developing anterior basal body, the Myc labeling is observed. Once the basal bodies are duplicated (above the dashed line), the Myc labeling is no longer detected, indicating that VFL3-1 is present only during an early step of the new basal body assembly.

**Fig. 9:**
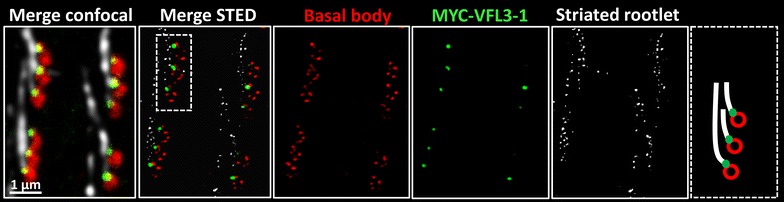
Localization of Myc-VFL3-1. Projections of confocal and corresponding STED sections performed on Myc-VFL3-1 expressing transformants stained by 1D5 (basal body) in *red*, anti-Myc (VFL3-1) in *green* and KD2 (striated rootlets) in *white*. The irregular KD labeling observed in confocal images appear as *dots* in STED imaging. The basal body microtubules visualized as *single dots* in confocal imaging appear as several puncta signals in STED imaging. VFL3-1 labeling localizes between the striated rootlet and the basal body microtubules to which they are attached. *Right panel*, scheme of the MYC-VFL3-1 localization at the basal body proximal region

The localization of VFL3-1 with respect to the basal bodies was analyzed by stimulated emission depletion super-resolution microscopy (STED). The triple labeling by 1D5 (basal bodies), KD2 (striated rootlet) and anti-Myc antibodies indicates that VFL3-1 localizes between the proximal part of the striated rootlets and the microtubule wall of the basal body (Fig. 9).

### Relationship between VFL3-A and Centrin 3

Depletion of VFL3-A and Centrin 3, both localized outside the basal bodies, yielded similar defects in the basal body anchoring [14]. To explore the relationships between VFL3-A and Centrin 3 which is required for the basal body movement toward the cell surface, we followed the fate of the fluorescence in GFP-Centrin 3 transformants after knockdown of VFL3-A. After two or three divisions, the GFP signal decreases in indicating that VFL3-A is required for the recruitment of Centrin 3 (Additional file 8: Figure S8). This could indicate that the assembly of the transient ALF structure which requires Centrin 3 for assembly could also be impaired by the VFL3 depletion.

## Discussion

One homolog of OFD1 is encoded in the *P. tetraurelia* genome, whereas four isoforms clustered into two families (*VFL3*-*A* and *VFL3*-*B*) encode proteins related to VFL3. The VFL3-A subfamily is more closely related to the *Chlamydomonas* protein. We show that OFD1 localizes at the proximal part of the transition zone and is required for the assembly of the basal body tip and for correct basal body anchoring at the cell surface. While the recruitments of OFD1 and Centrin 2 proceed independently, the localization/stability of OFD1 and FOR20 at the basal body are interdependent. The knock down of the VFL3-B family which encodes proteins permanently associated with the basal bodies, did not induce any phenotype. In contrast, the VFL3-A proteins are shown to be required for both the correct positioning of the basal body at the cell surface and the regular organization of its appendages which mark its rotational asymmetry. Contrary to the effect of OFD1 depletion, the non-anchored basal bodies display a fully organized distal part. The VFL3-1 protein localizes transiently, at an early stage of the basal body duplication process, to the proximal region of the parental basal body, between the striated rootlet and the microtubules to which it is associated. These results suggest a role of VFL3-A as an extrinsic factor controlling the basal body rotational polarity. The anchoring defect could be due to the loss of this polarity, which specifies the sites of assembly of the different structures which guide the movement of the basal body toward the cell surface.

### A role of OFD1 in motile cilia anchoring process

We have shown that OFD1, which localizes at the distal part of the ciliated and unciliated basal bodies, is recruited early during the formation of the basal body. Its loss leads to a defective transition zone and prevents basal body anchoring at the cell surface. This is in agreement with the known function of OFD1 in the assembly of the primary cilium in mammalian cells. Associated to the distal ends of both procentrioles and centrioles, it acts to build the distal appendages [16] that help to anchor the mother centriole to the ciliary vesicle during the intracytoplasmic ciliogenesis process. Our results do not fit observations in zebrafish which showed that *OFD1* invalidation does not completely prevent axoneme extension of the Kupffer’s vesicle cilia. In this case however, it was considered that only a fraction of OFD1 protein was depleted [40]. No clear defect of basal body docking was either observed in brain cells of mutant mice carrying a deletion of exons 4 and 5 of *OFD1* [41]. However a cDNA generated by the deletion [42] encoded a 106 amino acids long protein which retained the TOF/LisH/conserved domain required for centrosomal targeting [18]. The expression of such a protein could thus partially complement the entire OFD1 loss. We cannot exclude the hypothesis that the anchoring process differs from one cellular type to another. Nevertheless, our results are consistent with the docking impairment observed in airways multiciliated cells of patients with OFD1 mutations (Thauvin-Robinet et al. 2013). They also indicate that OFD1 is required in the basal body anchoring process which involves a direct docking at the cell surface.

We localized OFD1 at the proximal part of the transition zone, between the ciliary membrane and the microtubule doublets. This result confirms the observations obtained through super-resolution analysis on primary and motile cilia [43]. After labeling by an anti-OFD1 antibody, a ring of ninefold symmetry was observed at the interface between basal body and the cilium, suggesting that OFD1 could be associated with the microtubule doublets at this interface. Upon OFD1 depletion, we also observed rare intracytoplasmic basal bodies containing microtubules extension beyond the abnormal transition zone. This observation provides further support to the hypothesis that OFD1 could control the elongation of the microtubules doublets [16].

The interdependence of OFD1 and FOR20 for their recruitment/stability at the basal bodies suggests that they could form a complex within the basal bodies. This is supported by our previous work [8] which showed that like OFD1, FOR20 is recruited early in the assembly of basal bodies, is localized between the axoneme and the ciliary membrane of the transition zone and remains stably associated to the cortex when the basal body disassembles. There is evidence that such a complex could be formed in association with OFIP at the centrosome and pericentriolar satellite in mammalian cells [19]. It is thus possible that this complex could also be formed during the basal body assembly, since OFIP is conserved in *Paramecium*. Surprisingly, whereas we previously showed that Centrin 2 is required for FOR20 recruitment, we observed that its loss does not prevent the localization of OFD1 at the basal body. One explanation for this discrepancy could be that, in the absence of Centrin 2, as previously shown for OFIP [19], FOR20 and OFD1 do not form a complex. In this case, the lack of both Centrin 2 and FOR20 would not influence the stability/recruitment of OFD1.

### VFL3-A: a conserved role in the development of basal body appendages and basal body positioning

We show here that the expression of the *VFL3*-*B* genes does not complement the inactivation of *VFL3*-*A*, indicating that the genes have different functions. A single gene of each family, *VFL3*-*A* and *VFL3*-*B,* is encoded in the genome of *P. caudatum*, a *Paramecium* species which did not undergo the last two whole-genome duplications observed in *P. tetraurelia* [36]. Two genes were also identified in the genome of *Tetrahymena thermophila* indicating that the *VFL3* gene duplication is at least as old as the origin of the Ciliates. One can thus suppose that after duplication, one copy of *VFL3* developed a function completely distinct of the ancestral gene in Ciliates. Another possibility is that several functions were originally performed by the ancestral gene and then have been independently retained in each isoform after sub-functionalization. In *Paramecium*, the inability of the basal bodies in the VFL3-A depleted cells to segregate, to dock at the cell surface as well as to assemble their appendages at the right position, is quite similar to the phenotypical defects observed in the VFL3 mutant of *Chlamydomonas* [20]. The genes of the *Paramecium* VFL3-A family have thus conserved at least one common ancestral function with the VFL3 gene of *Chlamydomonas*. Further analyses will be required to understand the function of the VFL3-B family.

While OFD1 depletion leads to defects in basal body tip assembly, VFL3-A depleted cells display abnormalities in the organization of their associated rootlets, i.e., striated rootlets and microtubular ribbons. In addition, VFL3 is also required for the recruitment of Centrin 3 and thus, although not demonstrated here, its depletion would most probably affect the assembly of the ALF, a transient Centrin 3 dependent structure [13]. All these appendages have been demonstrated to play a crucial role in basal body positioning, i.e., basal body segregation and tilting [39] and required for the basal anchoring at the cell surface These data strongly suggest that abnormalities of the system of rootlets result in basal body segregation and anchoring defects observed during the VFL3 depletion. This is in agreement with the conclusion of Wright et al. [20] concerning the effect of the VFL3 mutation in *Chlamydomonas* and with studies in *Tetrahymena* which indicate that the DisAp mutation which affect the length of the striated fibers cause defect in basal body orientation [44, 45].

### VFL3-A an actor in the establishment of the basal body rotational asymmetry

Among the different phenotypical abnormalities which accompany the anchoring defect in VFL3-depleted cells, the ability of basal bodies to assemble several striated rootlets is the most surprising. The association of single striated rootlet per basal body is one character shared by all Ciliates. In all species, this rootlet has the same orientation with respect to the cell polarity and the direction of the ciliary beating [46, 47], and the same function of anchoring the basal bodies to the cell surface [37, 48, 49]. Together with two other rootlets, the postciliary and transverse microtubules ribbons, the striated rootlet marks the basal body rotational asymmetry revealing the specificity of each of the nine microtubule triplets [50]. In wild-type cells, the asymmetry of the basal body and associated rootlets is strictly transmitted from one generation to the next during basal body duplication [51]. The fact that several striated rootlets may be associated with basal bodies after VFL3-A depletion strongly suggests that this protein could be required for the acquisition of triplet specification allowing the establishment of rotational asymmetry.

The mechanisms of establishment of the rotational asymmetry are still unknown and remain an open debate: does this asymmetry result from an intrinsic property of the basal body/centriolar structure or is it externally imposed by the environment? One of these factors could be the mispositioning of the nascent basal body observed during VFL3-A depletion. The fact that such abnormalities are not observed after Centrin 3 depletion, which also generates mispositioned fully assembled basal bodies [13], suggests that the mispositioning per se does not induce a loss of polarity.

Two markers which localize asymmetrically within the basal body lumen in *Chlamydomonas* reveal an intrinsic asymmetry of the basal bodies: the acorn, a complex structure which attaches to specific triplets [52] and VFL1, a molecule which localizes near a subset of the microtubules triplets that faces one of its associated fibers [53]. We show here for the first time that a protein required for the expression of this asymmetry, VFL3, localized transiently outside the basal body during assembly of the neoformed one. This result indicates that the asymmetry is at least partially imposed by an external factor. VFL3-A could be a crucial actor in the asymmetry by allowing the recruitment of external or internal proteins, such as VFL1, which induce a specification of the microtubular triplets of the basal body in *Chlamydomonas* [53]. Further experiments on the respective roles of VFL3 and VFL1 in the establishment of the circumferential asymmetry of the basal bodies are needed to analyze these mechanisms.

## Concluding remarks

The present study extends our knowledge on the molecular mechanisms involved in the basal body anchoring/positioning process in the multiciliated cell *P. tetraurelia.* We show that this process depends on the function of the OFD1 and VFL3-A protein families. Their loss prevents insertion and orientation of the basal bodies along the cellular antero-posterior axis and induces an accumulation of subcortical basal bodies. Although the phenotypical defects are quite similar, the underlying mechanisms are different and highlight the importance of the assembly of both distal and proximal structures of the basal body in the orientation and the docking process at the cell surface. In addition, our results suggest that the mispositioning of the basal bodies at the cell surface might result of the loss of the rotational asymmetry of their microtubular triplets.

## Additional files

**Additional file 1: Figure S1.** Nucleotidic sequences of VFL3 genes. The entire nucleotidic sequence of each VFL3 gene is indicated. In red are the sequences cloned in the L4440 vector for the silencing experiments. In blue are the sequences used as probes for Northern analyses.

**Additional file 2: Figure S2.** A phylogenetic analysis of VFL3. Cladogram showing the relationships between the four *Paramecium tetraurelia* VFL3 isoforms (VFL3-1, VFL3-2, VFL3-3 and VFL3-4) and their homologs in others species. The evolutionary history, based on the alignment of the conserved N-terminal parts of the proteins was inferred by using the one click mode at Phylogeny.fr [54]; Boostrap value are displayed as probability. The scale is in the units of the number of amino acid substitutions per site. H.sapiens: ENSP00000263284; T.thermophila-1: XP_001015880; T.thermophila-2: XP_001012873; X. laevis: NP_001089598; D.rerio: XP_005161271; C.reinhardtii: XP_001695308; S.mediterranea: SMU15034611; P.caudatum-1: PCAUDP15713; P.caudatum-2: PCAUDP02708; P.tetraurelia: VFL3-1: GSPATP00031209001; VFL3-2: GSPATP00018236001; VFL3-3: VFL3-3 GSPATP00013051001; VFL3-4: GSPATP00008368001.

**Additional file 3: Figure S3.** OFD1 are evolutionary conserved proteins. Alignment of the N-terminal part of the *Paramecium tetraurelia* OFD1 protein with OFD1 proteins of other species. H.sapiens: NP_003602; T.thermophila: XP_001007171; P.tetraurelia: GSPATP00001073001; X.laevis: XP_018102518; D.rerio: XP_009303289.

**Additional file 4: Figure S4.** Decrease of the GFP signal in GFP-OFD1 transformants after OFD1 depletion. The efficiency of the OFD1 RNAi vector to inactivate the corresponding gene was evaluated by following the fluorescence in GFP-OFD1 expressing cells upon inactivation. The cell is representative of n>25. Projections of confocal sections passing through the dorsal surface of transformant expressing GFP-OFD1 after divisions upon inactivation (A) with the control vector or (B) with the vector specific of *OFD1*. Red: basal bodies labelled with 1D5; green: GFP-OFD1. After divisions parental basal bodies and new basal bodies assembled during the inactivation are mixed within the rows. In the control cell (A), GFP-OFD1 localizes at all basal bodies (arrows in the insets point corresponding basal bodies) indicating that the tagged protein is continuously expressed. Upon inactivation with the OFD1 specific vector (B), only a fraction of basal bodies are associated with the GFP labelling. These basal bodies correspond to those present at the cell surface before the RNAi, as demonstrated by their regular alignment into antero-posterior rows. By contrast new basal bodies, assembled in erratic localisations, are not associated with GFP-labelling (arrows in B). This correlation between the mislocalisation, specific for OFD1 depletion, and the absence of labelling indicates that the expression of the GFP-OFD1 is affected (Arrowheads in the inset).

**Additional file 5: Figure S5.** Relationships of OFD1 with Centrin 2 and FOR20. Images are projection of confocal sections In all experiments, the GFP signal was observed on cells labeled by 1D5 (red). In the control cells all the basal bodies retained the GFP signal. Inactivation of OFD1 in GFP-Centrin2 expressing cells and inactivation of Centrin2 in GFP-OFD1 expressing cells show that the GFP signal is retained in all basal bodies (arrows) after 2 divisions upon inactivation. In contrast, inactivation of FOR20 in GFP-OFD1 expressing cells and inactivation of OFD1 in GFP-FOR20 expressing cells reveal a reduction of the GFP labelling on numerous basal bodies (arrows). As explain in figure S4, the coexistence of basal bodies brightly labelled, as in control cells, and basal bodies harboring a reduced signal is due to the coexistence in the same cell of parental basal bodies and new basal bodies assembled during the inactivation. In inactivated cells, the misorientation and mispositioning of basal bodies with regard to the cell surface explain that the GFP and 1D5 labelling do not strictly overlap since FOR20 and OFD1 are associated with the distal part of the basal bodies.

**Additional file 6: Figure S6.** *VFL3-A* and *VFL3-B* RNAi efficiencies. Efficiency of the *VFL3-A* and *VFL3-B* RNAi vectors to inactivate their target sequences was tested by northern Blots. RNA extracted from cells inactivated for *VFL3-A* family (*VFL3-1* and *VFL3-2* genes), *VFL3-B* family (*VFL3-3* and *VFL3-4* genes) and *ND7* (a gene involved in trichocyst discharge used as control) were transferred on blots and hybridize with ^32^P-labelled probes. Details for all the probes are in Methods. Hybridization signals were normalized using 17S rRNA. Numbers indicate the rate of target expression in RNAi-treated cells, relative to the control. RNAi triggered either by VFL3-1 or VFL3-2 (VFL3-A family) results in ~75% decrease in the total amount of VFL3-1 and VFL3-2 mRNA but does not reduce VFL3-3 and VFL3-4 (VFL3-B family) mRNA. RNAi triggered VFL3-3 result in a 63% decrease in the total amount of VFL3-3 mRNA but not reduce VFL3-1 and VFL3-2 (VFL3-A family) indicating that the probes are specific of each family. The weak signal observed with the VFL3-4 probe indicates that the *VFL3-4* gene is poorly expressed.

**Additional file 7: Figure S7.** Localization of Myc-VFL3-3. Projection of confocal sections through transformants expressing Myc-VFL3-3 fixed and labelles with 1D5 (basal body) and anti-Myc antibody (Myc-VFL3-3). The Myc signal colocalizes with the 1D5 labelling at all basal bodies.

**Additional file 8: Figure S8.** Relationship between VFL3-A and Centrin 3. Projections of confocal section performed on cells expressing GFP-Centrin3 inactivated by the VFL3 specific vector (left) or by the cpntrol vector (right) on cells labeled by 1D5 (red). In the control cell, parental and newly assembled basal bodies retained the GFP signal. Inactivation of the *VFL3-A* isoforms in GFP-Centrin 3 expressing cells induces a reduction of the GFP signal in the newly assembled basal bodies (arrows).

## Abbreviations

ALF: anterior left filament
FOR20: FOP-related protein of 20 kDa
OFD1: oro-facial-digital syndrome 1 protein
OFIP: FOR20 interacting protein
VFL3: variable flagellar number mutant 3
VFL1: variable flagellar number mutant 1
Immuno EM: immunoelectron microscopy
WT: wild type

## References

[1] Sorokin SP (1968). Reconstructions of centriole formation and ciliogenesis in mammalian lungs. J Cell Sci.

[2] Sorokin S (1962). Centrioles and the formation of rudimentary cilia by fibroblasts and smooth muscle cells. J Cell Biol.

[3] Hagiwara H, Shibasaki S, Ohwada N (1992). Ciliogenesis in the human oviduct epithelium during the normal menstrual cycle. J Electron Microsc (Tokyo).

[4] Park TJ, Mitchell BJ, Abitua PB, Kintner C, Wallingford JB (2008). Dishevelled controls apical docking and planar polarization of basal bodies in ciliated epithelial cells. Nat Genet.

[5] Burke MC, Li F-Q, Cyge B, Arashiro T, Brechbuhl HM, Chen X (2014). Chibby promotes ciliary vesicle formation and basal body docking during airway cell differentiation. J Cell Biol.

[6] Wei Q, Ling K, Hu J (2015). The essential roles of transition fibers in the context of cilia. Curr Opin Cell Biol.

[7] Dippell RV (1968). The development of basal bodies in paramecium. Proc Natl Acad Sci USA.

[8] Aubusson-Fleury A, Lemullois M, de Loubresse NG, Laligné C, Cohen J, Rosnet O (2012). The conserved centrosomal protein FOR20 is required for assembly of the transition zone and basal body docking at the cell surface. J Cell Sci.

[9] Dute R, Kung C (1978). Ultrastructure of the proximal region of somatic cilia in Paramecium tetraurelia. J Cell Biol.

[10] Aubusson-Fleury A, Lemullois M, Bengueddach H, Abdallah S, Shi L, Cohen J (2015). Transition zone: the sequential assembly of its components parallels its dual role in basal body anchoring and ciliary function. Cilia.

[11] Iftode F, Cohen J, Ruiz F, Rueda AT, Chen-Shan L, Adoutte A (1989). Development of surface pattern during division in Paramecium. I. mapping of duplication and reorganization of cortical cytoskeletal structures in the wild type. Development.

[12] Iftode F, Addoute A, Fleury A. The surface pattern of paramecium tetraurelia in interphase: an electron microscopic study of basal body variability, connections with associated ribbons and their epiplasmic environment. Eur J Protistol. 1996.

[13] Jerka-Dziadosz M, Koll F, Włoga D, Gogendeau D, Garreau de Loubresse N, Ruiz F (2013). A Centrin 3-dependent, transient, appendage of the mother basal body guides the positioning of the daughter basal body in Paramecium. Protist.

[14] Ruiz F, Garreau de Loubresse N, Klotz C, Beisson J, Koll F (2005). Centrin deficiency in Paramecium affects the geometry of basal-body duplication. Curr Biol.

[15] Ferrante MI, Giorgio G, Feather SA, Bulfone A, Wright V, Ghiani M (2001). Identification of the gene for oral-facial-digital type I syndrome. Am J Hum Genet.

[16] Singla V, Romaguera-Ros M, Garcia-Verdugo JM, Reiter JF (2010). Ofd1, a human disease gene, regulates the length and distal structure of centrioles. Dev Cell.

[17] Azimzadeh J, Nacry P, Christodoulidou A, Drevensek S, Camilleri C, Amiour N (2008). Arabidopsis TONNEAU1 proteins are essential for preprophase band formation and interact with centrin. Plant Cell.

[18] Sedjaï F, Acquaviva C, Chevrier V, Chauvin J-P, Coppin E, Aouane A (2010). Control of ciliogenesis by FOR20, a novel centrosome and pericentriolar satellite protein. J Cell Sci.

[19] Chevrier V, Bruel A-L, Dam TJPV, Franco B, Scalzo ML, Lembo F (2016). OFIP/KIAA0753 forms a complex with OFD1 and FOR20 at pericentriolar satellites and centrosomes and is mutated in one individual with oral-facial-digital syndrome. Hum Mol Genet.

[20] Wright RL, Chojnacki B, Jarvik JW (1983). Abnormal basal-body number, location, and orientation in a striated fiber-defective mutant of *Chlamydomonas reinhardtii*. J Cell Biol.

[21] Gupta GD, Coyaud É, Gonçalves J, Mojarad BA, Liu Y, Wu Q (2015). A dynamic protein interaction landscape of the human centrosome-cilium interface. Cell.

[22] Jakobsen L, Vanselow K, Skogs M, Toyoda Y, Lundberg E, Poser I (2011). Novel asymmetrically localizing components of human centrosomes identified by complementary proteomics methods. EMBO J.

[23] Skouri F, Cohen J (1997). Genetic approach to regulated exocytosis using functional complementation in Paramecium: identification of the ND7 gene required for membrane fusion. Mol Biol Cell.

[24] Sonneborn TM, Prescott DM (1970). Chapter 12 methods in Paramecium research. Methods in cell biology.

[25] Timmons L, Fire A (1998). Specific interference by ingested dsRNA. Nature.

[26] Arnaiz O, Cain S, Cohen J, Sperling L (2007). ParameciumDB: a community resource that integrates the Paramecium tetraurelia genome sequence with genetic data. Nucleic Acids Res.

[27] Aury J-M, Jaillon O, Duret L, Noel B, Jubin C, Porcel BM (2006). Global trends of whole-genome duplications revealed by the ciliate *Paramecium tetraurelia*. Nature.

[28] Lepère G, Bétermier M, Meyer E, Duharcourt S (2008). Maternal noncoding transcripts antagonize the targeting of DNA elimination by scanRNAs in *Paramecium tetraurelia*. Genes Dev.

[29] Galvani A, Sperling L (2002). RNA interference by feeding in Paramecium. Trends Genet.

[30] Beisson J, Bétermier M, Bré MH, Cohen J, Duharcourt S, Duret L, et al. Immunocytochemistry of Paramecium cytoskeletal structures. Cold Spring Harb Protoc. 2010 10.1101/pdb.prot5365 20150124

[31] Wehland J, Weber K (1987). Turnover of the carboxy-terminal tyrosine of alpha-tubulin and means of reaching elevated levels of detyrosination in living cells. J Cell Sci.

[32] Janke C, Bulinski JC (2011). Post-translational regulation of the microtubule cytoskeleton: mechanisms and functions. Nat Rev Mol Cell Biol.

[33] Nahon P, Coffe G, Guyader H, Darmanaden-Delorme J, Jeanmaire-Wolf R, Clerot JC (1993). Identification of the epiplasmins, a new set of cortical proteins of the membrane cytoskeleton in Paramecium. J Cell Sci.

[34] Sperling L, Keryer G, Ruiz F, Beisson J (1991). Cortical morphogenesis in Paramecium: a transcellular wave of protein phosphorylation involved in ciliary rootlet disassembly. Dev Biol.

[35] Callen A-M, Adoutte A, Andrew JM, Baroin-Tourancheau A, Bré M-H, Ruiz PC (1994). Isolation and characterization of libraries of monoclonal antibodies directed against various forms of tubulin in Paramecium. Biol Cell.

[36] McGrath CL, Gout J-F, Doak TG, Yanagi A, Lynch M (2014). Insights into three whole-genome duplications gleaned from the *Paramecium caudatum* genome sequence. Genetics.

[37] Aubusson-Fleury A, Bricheux G, Damaj R, Lemullois M, Coffe G, Donnadieu F (2013). Epiplasmins and epiplasm in paramecium: the building of a submembraneous cytoskeleton. Protist.

[38] Romero MR, Torres A (1993). Cortical development associated with conjugation of Paramecium. Development.

[39] Iftode F, Fleury-Aubusson A (2003). Structural inheritance in Paramecium: ultrastructural evidence for basal body and associated rootlets polarity transmission through binary fission. Biol Cell.

[40] Ferrante MI, Romio L, Castro S, Collins JE, Goulding DA, Stemple DL (2009). Convergent extension movements and ciliary function are mediated by ofd1, a zebrafish orthologue of the human oral-facial-digital type 1 syndrome gene. Hum Mol Genet.

[41] D’Angelo A, De Angelis A, Avallone B, Piscopo I, Tammaro R, Studer M (2012). Ofd1 controls dorso-ventral patterning and axoneme elongation during embryonic brain development. PLoS ONE.

[42] Ferrante MI, Zullo A, Barra A, Bimonte S, Messaddeq N, Studer M (2006). Oral-facial-digital type I protein is required for primary cilia formation and left-right axis specification. Nat Genet.

[43] Lee YL, Santé J, Comerci CJ, Cyge B, Menezes LF, Li F-Q (2014). Cby1 promotes Ahi1 recruitment to a ring-shaped domain at the centriole–cilium interface and facilitates proper cilium formation and function. Mol Biol Cell.

[44] Galati DF, Bonney S, Kronenberg Z, Clarissa C, Yandell M, Elde NC (2014). DisAp-dependent striated fiber elongation is required to organize ciliary arrays. J Cell Biol.

[45] Jerka-Dziadosz M, Jenkins LM, Nelsen EM, Williams NE, Jaeckel-Williams R, Frankel J (1995). Cellular Polarity in ciliates: persistence of global polarity in a disorganized mutant of *Tetrahymena thermophila* that disrupts cytoskeletal organization. Dev Biol.

[46] Lynn DH (1981). The Organization and evolution of microtubular organelles in ciliated protozoa. Biol Rev.

[47] Sleigh MA, Silvester NR (1983). Anchorage functions of the basal apparatus of cilia. J Submicrosc Cytol.

[48] Peck RK (1977). The ultrastructure of the somatic cortex of Pseudomicrothorax dubius: structure and function of the epiplasm in ciliated protozoa. J Cell Sci.

[49] Williams NE, Vaudaux PE, Skriver L (1979). Cytoskeletal proteins of the cell surface in Tetrahymena I. Identification and localization of major proteins. Exp Cell Res.

[50] Grain J (1969). Le cinétosome et ses dérivés chez les ciliés. Ann Biol.

[51] Beisson J, Sonneborn TM (1965). Cytoplasmic inheritance of the organization of the cell cortex in *Paramecium aurelia*. Proc Natl Acad Sci USA.

[52] Geimer S, Melkonian M (2004). The ultrastructure of the *Chlamydomonas reinhardtii* basal apparatus: identification of an early marker of radial asymmetry inherent in the basal body. J Cell Sci.

[53] Silflow CD, LaVoie M, Tam LW, Tousey S, Sanders M, Wu W (2001). The Vfl1 Protein in Chlamydomonas localizes in a rotationally asymmetric pattern at the distal ends of the basal bodies. J Cell Biol.

[54] Dereeper A, Guignon V, Blanc G, Audic S, Buffet S, Chevenet F (2008). Phylogeny.fr: robust phylogenetic analysis for the non-specialist. Nucleic Acids Res.

