# Supplementary material for: Basal body positioning and anchoring in the multiciliated cell Paramecium tetraurelia: roles of OFD1 and VFL3
Source: Cilia. 2017 Mar 30;6:6. doi: 10.1186/s13630-017-0050-z (PMC5374602; doi:10.1186/s13630-017-0050-z)
Supplement: Supplementary file 1 — Additional file 1: Figure S1. Nucleotidic sequences of VFL3 genes. The entire nucleotidic sequence of each VFL3 gene is indicated. In red are the sequences cloned in the L4440 vector for the silencing experiments. In blue are the sequences used as probes for Northern analyses. [file 13630_2017_50_MOESM1_ESM.pdf]

>Vfl3-1

ATGGAAGATCTCCTTGATAT**CTGTGTTGAACGCTAATTTCATGATGTCAATTACATTATCAACATTAATGCAAA
TGTTGAGACTCTAAATATTGAAATTGAATCGAAACAATCTGGTGACTCTTGGATTGCCAATTTCTAAGCTCAA
TATATTGAAGATATTGCATCAAAAACTGGAAATTATAAGAAATATGCCACGTTCATAAAGATGCTCTAGTCA
GCGTTGAATAATTAAACTGAGACCGTGTATATCGACATCCTGACCTATTAAGATTTGGAGCAAATAAAAAAT
AAGAGGTCCAATAAATAACCAACTCAAAACCTAGCTCCTAATAATAAACGCTACCTTATCCTTAGTTATGTTG
TAGAATTTGATAAAGTGCATTATCCTTTACCATTAAATTTTAATGAATAACCAAATTTATAATAAATGAAGAA
TACGATTATTAGATTAAGGAAAGAAAATGAATCATTGCATAAAAATCTCTAATTTTACAAGGATTAAAAACG
AAACGAATCCAACCCTTCAATTTTGTTGAATGAATAAATAAGAGAATTAGAAATGTTAAGAAACATCATTGA
TCAAAAGGAAAGTGAAATTTTAGAATTAAAAAATATGAATCATCAATATTCCAATA**CAATGATAAATAGATA
AGAATACAATGACCTAAGATCAAAATATCTATAAAGTGAAAATACAAATTCAGAATTATCAAAAAAAATGATC
GAATTTGAAGACTATTTACAATAATTGATAGATGAAAATGTAATACTGAAGAACTAAGATAAAAAGAATAAAT
AAAGAATAAACTCATTGGAATCTGAATTACAGTAAACTCTAAATAAATCAAAGTCCAAATATAATTCGAATCTT
TCTCGATCTCCGTCTGTTTCAAAATCTAACAATAAATAAAAAAACTAACTCAACACCAGTAATATACCTTCCAAA
AGAAAAAGCATTGAGAAAACAACTCCAATTAGAgtaattttcatattaatatcattagTTGCGAAAAGATAGTTTGGATA
GTGATACAGAGTCTTCTTACAGAAGAAAATCCAATTCTAAAAGAACTGAATAAAGTCCCTCAGCAAGATCAAG
TTCATCAAAAAAAAAGTCATTCTAACCTAAACAATCCAATTCAAAACTACAAAAAAATAAAAATGTTATTAAACC
ATCAGATTCGAATAAGTTCGAGGATTCAGAAGAATAGCGTTTAATTAAAAGATTAAGAGATTTAAGAGAAAAG
AATAAAGAGAATACAATCATAACTACTCCTTAAAGCAAAATCGAAGCTACGACTGAAGATTTATAGAGTATAG
ATGTAAGACTCAATAAATTAAATACATTACTGTAATTAGCTAAAAATTAATGA

>Vfl3-2

ATGGAAGATCTCCTTGATATAAATGTGGAAAGAAACTTTCATGACATCACTTACATCATAAATATTAATGCCAA
TACTGAAATGCTGACAATAGATGTAGAATCGAAGTAATCGGGAGATTCTTGGATCGCAAACTTTTAAGCATCCT
ACATCGAGGAAATCACATCCAAGACTGGGAATTATAAGAAATACCCAACATTTCTCAAGATGTTGTAATCTGCA
ATAAAAAATCAGACAGACACCGTCTATATTGACATTCTCACATTCTAAGATCTAGAGCAAATCAAAAACAAAAG
ACCCAAATAAAATTAATAGCAAAATCTGGTCCCAAATAATAAACGTTATTTGATTCTTAGTTACATAGTTGAATT
TGATAAAGTTCATTATCCATTACCTCTCAATTTTAATGAGCAGCCCAACATTTAGCAAATGAAGAATACCATTAT
AAGGTTGAGAAAGGAGAATGAACAATAAGCAAAATAATTGACAACGCTCTTAGAGTCTAAGAAAAACGAAGT
TAATCCTTTGGAGGTGATAAATGAACAGAACAAGGAAATCGAGTTTTTAAAAAATCTATTGGAATAAAAAGAT
AGAGACATTTTGGAACTCAAAAACATAGTGCATCAATAGTCTTCAACAACTGTGAACAAATAGGAGTATATTG
AATTGAGATCAAAATACTTGCAAAGTGAAAATACAAATGCAGAAATCACTAAGAAAGTGATTGAATTAGAAGA
TTGTCTCCAGTAAATTATAGATGAAAATCATCTATTAAAAAACTAAGATAAAAAAAATAAATAGAGGATAACAT
CTTTGGAATAGGAACTATAATAAACTCTAACAAGATCTAAAACAAAATTGCAACCGAACATTTCAAGGTCTCCT
TCAACTTCTAAATCCTCAAATCAATTCAAGGTTTCAAGTAGCATTAACAAAGTATTATCGAAAAAGAAAAGTGC
AGAGAAAACAACCCCCATCAGAgtataatttattttaatatatttagATTAGAAAAGAGAGTTTTGATAGTGATACTGAG
TCCTCTTACAGACGTAAATCAAATTCTAAAAGAACAGATCAGAGTCCATCTGCAAGGTCGAATTCTTCTCGCAA
AACATCTTAAAAATTGAAACAATCAAATTCAAATTTATTCAAAAAATAAAAGCAAACGACAACTAATGAATCCA
GAAAATTTGAAGATCCTGAGGAATAGAGACTTTTAAAAAGATTGAGGGATTTGAGAGAAAAAAATAAGGAAA
ATACAATAGTAACTACTCCATCTAATAAAATGGAAGCCAGTTGTGAGGATTTGCATAGTATTGATGAAAGATTA
AACAAATTAAACAATCTATTGCAAAGAGCAAAAAATTAATGA

>VFL3-3

ATGCTAGATAATCCAAAAATATAATTAGAAACAGACATCATTCTTCAAGGAATGGAATACGTCATTAGCATGCA
AGCTAGTGATCATTTGCTgtatgcataattaatacccttagTTATATAGAATTAGAATCTAAATATGAACCTCAGATCTG
GAAAAATACTTACACCATTGATTATATTGAAGAACTTACTCGCAAGACAGGAAACCCAAAAAAATTTAACATAT
TTCTCTCCATGTTATAAACTGCATTACAAAAAACTAACGAGAATgtattatataatctaaatagGTTTTATTTGAAATTT

TGACGTATCAAGATCTTGAGTAATAGAAATCATAAGATCAAAGCAATCTTTCTAGAACATCATCCAATAATTAG
AAAGTCAATAAACGATATCTGATTTTGTCGTATTAAACTGATTTTGAAAAAATTCATTACCCTTTGGCCTTAAAT
TATGAAGAATAAATTGAAAACTCTAGATTAATGACCAAAATACAGAATCTAAAAACAGAACTCTTTGATTACAA
ACTCTAAAAATCAAATGATGCAGATTTCTAGGCTTCGAATTCATTTTCCAAAAGCAAAAGGGATATTTAAACTC
CTGATTCTCTAGTGAATCAAAATGAATTATTGAAAGCCAAAGTAAAGAGATTGGAAGAAGCTTTGACTTAAAA
AAAGGGGGCAGTTGAAGTAGATTAATTAGTTCGAGATAATGAAGACTTGTAAAATATGTTTACTACATCTAAA
TTATTATATGAAGACAAAATCTAAAAGTTAGAACAACAACTCAATTAAAAAAGCACAGAAATTGTCATGCAAAT
GGCTGAAgtacatataaagttaataaagattagATAAATGCTTTTAAGAAGGAGTTGACACAATTGATTGGCAAAGTTG
AAATGGACAGTAGAATCAAAGATCATATCAGATTAATGAATGAGGAGGAGGAAAGCAAATTAGTGAAAGCCT
AAAAAACAATATAAAAATACGAAAAAGAAGTGGAAGGCCTTAATTAgtatgtaattgataaaatcatagATAAATAGA
TTCGTTAAAGAAATAAGACACAGTCTAAAAAAGAAGAATAAATCAATTGGAGACTGAACTATCTTAATCGATG
AAAAAGTTTAGTTACAAGGGAGTTACTGATAGGCTTTATTCACCCTACAGTAATCATTCCAATGGAAGTAGGAA
GTCTAATTATTCTGTTCCAAATAGATTAAACTCGAATAATTCATCTGGGAATGCTTCCCCTAATGTAAGACAAGC
TTCTCCAGCTTTAAGCAAGAATTCGCGTACTTCCACTTAAAATTCAgtaaaatcatattcaaaattagGCTGCCTATCAG
AAACCGAAGTTCTATAACACTTCTCCAATGAATAAACAGCCCATTTAGAAGAAACCCTCTCCGGCAAGACCATA
ATAATAGATGAGACCTACTCCCAATAGGGCATCTCCAGTTGCTTAATAGAAACCTTCTCCTGGGAGAAATAATA
TCTTTAGATAGCCATCTCCTCCAAGATAAAACTACTAACAACAAAAGATTCAGCCATAACAATAAGTGAAATAA
AGGACTAATCAATCTGCTAATAATTATAAGTAGCCAATGAAGCAGGTGCATCAATATGAAAGGAAGTCTGGAA
ATGTGACGGATCTGGATAATAAAATAAATAATTTAAAAAATATTTTATAAAGAGCGAAAAGATGA

>VFL3-4

ATGCTTGATAATCCAAAAATATAACTAAAAACAGAACTAACATTACTAGGAATGGAATACTTAATCAATATTCA
AGCTAATGATCATTTACTgtatatatatattatactttagTTATATAGAATTAGAATCGAAATATGCACCCTAAATCTGG
AAAAATACTTACACTATTGATTATATTGAAGAGCTTACTCGTAAAACAGGAAATCAGAAAAAATTCAATGTATT
TCTTTCCATGTTATAAACTGCAATATAAAAAACCAATGAAAATgtattaaaatatattttaattagATATTTTTTGAAATT
TTGACATACCAAGATCTAGA**G**AATCTCAAATAATAGAAATAATAAAATTAAAGTCATATTTCTAGAACGTCATC
AACGAATTCAAAAATCAATAAAAGATATCTCATCTTGTCATATAAGAATGATTTAGAAAAGGTTCATTATCCTTT
AGCCTTAAATTATGAAGAACATATTGAAAACTCAAGATTAATGACTAAAATCTAGAATCTAAAAACCGAATTAT
TAGAGCATAAATCATAAAAATAGAATGACTCAGAATTTTAAGTTTCGAATTTAATTTCCATTAGTAAAAGGGAT
AATTAAACATTCGATTCCTTAGTGAATTAAAATGAATTATTGAAAGCAAAGGCCAAGAGATTGGAAGAGGCTT
TGACCCAAAAAAAAGGGGCAGTTGAAGTGGATTAATTGATTAGAGATAATGAGGACTTATAAAATTTGTTAAA
TACATCCAAATTGTTATATGAAGAGAAGATCTAGAAGTTAGAACAATAGATAGATTTGAAAAGTACAGAAATC
GTTCT**G**TAAATGTCTGAAgtacttataatgaaaataaagattagATAAATGCATTTAAGAAAGAGTTATCTAGATTGATT
GGCTAAGTAGAGATGGATAATCATATCAAAGAGAAAATCAAACTTATGAATGAGGATGAGGAAAGTAAATTG
GTAAAAGCATAGAAAACTATTCAAAAATATGAAAAAGAAGTGGAAGCTCTTATATAgtatgtaattgataaaatcatag
ATAAATAGATTCATTAAAGAAATAAGATGCAGTACAAAAGAGAAAAATAAATTAATTGGAGACCGAACTATCT
TAGTCAATGAAAAGGTTTAGTTACAAAGGAGTTACTGACAGACTTTATTCTTCATATAGTAACCACTCTAATGCT
AGCAAAAAATCGAATTACTCAGTTCCGAAAAGAGAGAATTCAAATAATTCATCTAGAAATGCATCTCCAAATGT
AAGGCAGACTTCTCCAATTTTAAATAAGAATTCACATTCTTCTGTTTAAAATTCAgtaagtatagacagtattttagTCTG
CAAATCAAAGACCTAAGTTTTATAACACTTCTCCAATGAATAAACAGCCAATTTAGAAGAAATCATCTCCAGCT
AGACCCCAACAATAGATTAAGCCTAATCCTATTAGGACATCTCCAATAACTTAATAGAAACCATCTCCTGGCAG
AAACAATATCTTTAAATAGCCTTCTCCTCCAAGATAATATAATTAACAACATAGAGTTTCTCAATTATAGTAGAC
TAAATAAAGGACAAATTAATCTGCTAATAATTATAAGTAACCTATCAAATAGGTACACCAATATGATTGGAAGT
CAGGGAATGTAACAGAACTTGACAATAAAATAAATAATTTAAAAAATATATTGTCGAAAGCTAAAAGGTGA
Data were obtained from three biologically independent experiments
