# Supplementary figures and images for: Basal body positioning and anchoring in the multiciliated cell Paramecium tetraurelia: roles of OFD1 and VFL3
Source: Cilia. 2017 Mar 30;6:6. doi: 10.1186/s13630-017-0050-z (PMC5374602; doi:10.1186/s13630-017-0050-z)

## Slide 1
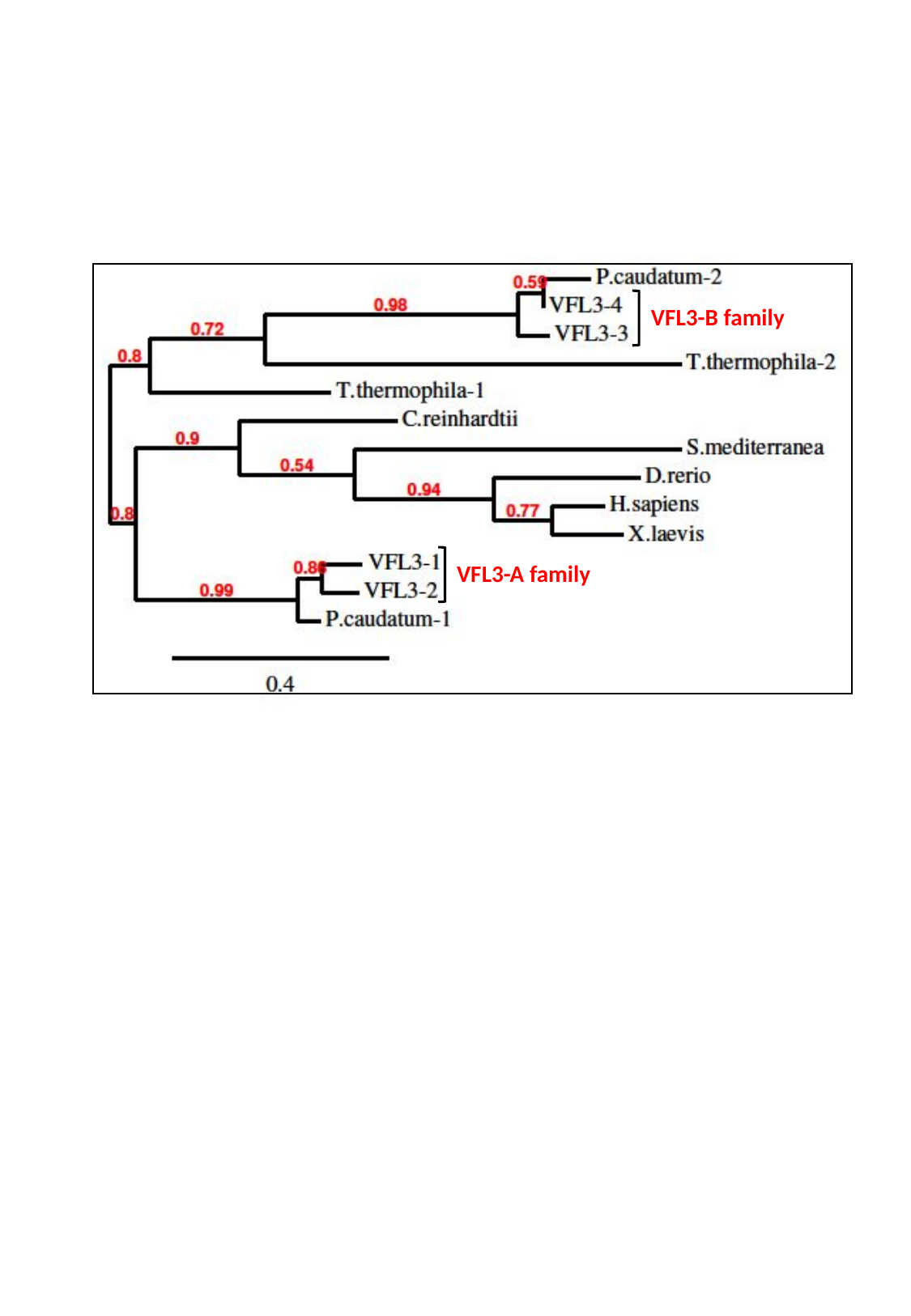

VFL3-B family
VFL3-A family

Supplement: Supplementary file 2 — Additional file 2: Figure S2. A phylogenetic analysis of VFL3. Cladogram showing the relationships between the four Paramecium tetraurelia VFL3 isoforms (VFL3-1, VFL3-2, VFL3-3 and VFL3-4) and their homologs in others species. The evolutionary history, based on the alignment of the conserved N-terminal parts of the proteins was inferred by using the one click mode at Phylogeny.fr [54]; Boostrap value are displayed as probability. The scale is in the units of the number of amino acid substitutions per site. H.sapiens: ENSP00000263284; T.thermophila-1: XP_001015880; T.thermophila-2: XP_001012873; X. laevis: NP_001089598; D.rerio: XP_005161271; C.reinhardtii: XP_001695308; S.mediterranea: SMU15034611; P.caudatum-1: PCAUDP15713; P.caudatum-2: PCAUDP02708; P.tetraurelia: VFL3-1: GSPATP00031209001; VFL3-2: GSPATP00018236001; VFL3-3: VFL3-3 GSPATP00013051001; VFL3-4: GSPATP00008368001. [file 13630_2017_50_MOESM2_ESM.pptx]

## Slide 1
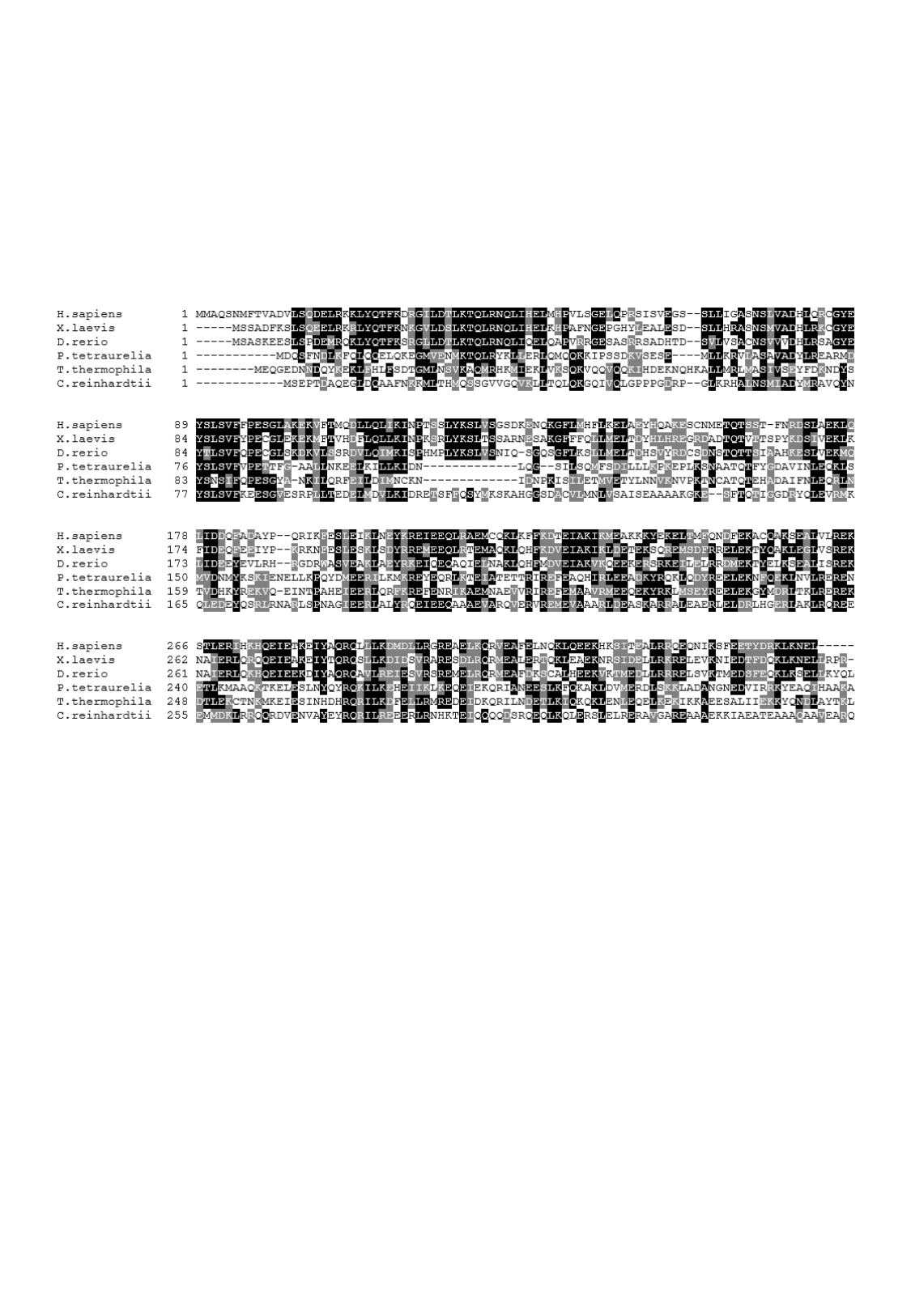

Supplement: Supplementary file 3 — Additional file 3: Figure S3. OFD1 are evolutionary conserved proteins. Alignment of the N-terminal part of the Paramecium tetraurelia OFD1 protein with OFD1 proteins of other species. H.sapiens: NP_003602; T.thermophila: XP_001007171; P.tetraurelia: GSPATP00001073001; X.laevis: XP_018102518; D.rerio: XP_009303289. [file 13630_2017_50_MOESM3_ESM.pptx]

## Slide 1
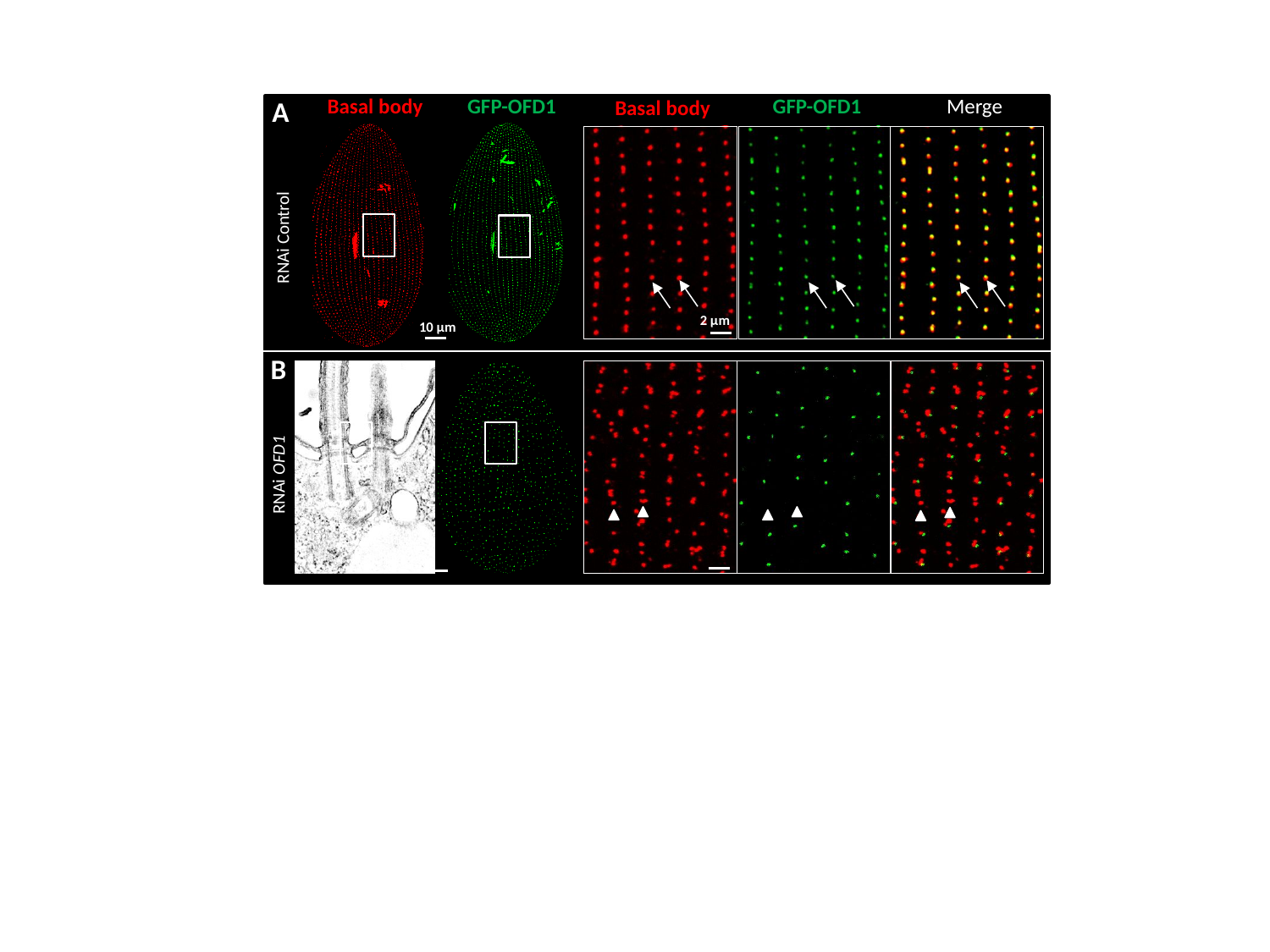

GFP-OFD1
Basal body
GFP-OFD1
Merge
Basal body
A
RNAi Control
2 µm
10 µm
B
RNAi OFD1

Supplement: Supplementary file 4 — Additional file 4: Figure S4. Decrease of the GFP signal in GFP-OFD1 transformants after OFD1 depletion. The efficiency of the OFD1 RNAi vector to inactivate the corresponding gene was evaluated by following the fluorescence in GFP-OFD1 expressing cells upon inactivation. The cell is representative of n>25. Projections of confocal sections passing through the dorsal surface of transformant expressing GFP-OFD1 after divisions upon inactivation (A) with the control vector or (B) with the vector specific of OFD1. Red: basal bodies labelled with 1D5; green: GFP-OFD1. After divisions parental basal bodies and new basal bodies assembled during the inactivation are mixed within the rows. In the control cell (A), GFP-OFD1 localizes at all basal bodies (arrows in the insets point corresponding basal bodies) indicating that the tagged protein is continuously expressed. Upon inactivation with the OFD1 specific vector (B), only a fraction of basal bodies are associated with the GFP labelling. These basal bodies correspond to those present at the cell surface before the RNAi, as demonstrated by their regular alignment into antero-posterior rows. By contrast new basal bodies, assembled in erratic localisations, are not associated with GFP-labelling (arrows in B). This correlation between the mislocalisation, specific for OFD1 depletion, and the absence of labelling indicates that the expression of the GFP-OFD1 is affected (Arrowheads in the inset). [file 13630_2017_50_MOESM4_ESM.pptx]

## Slide 1
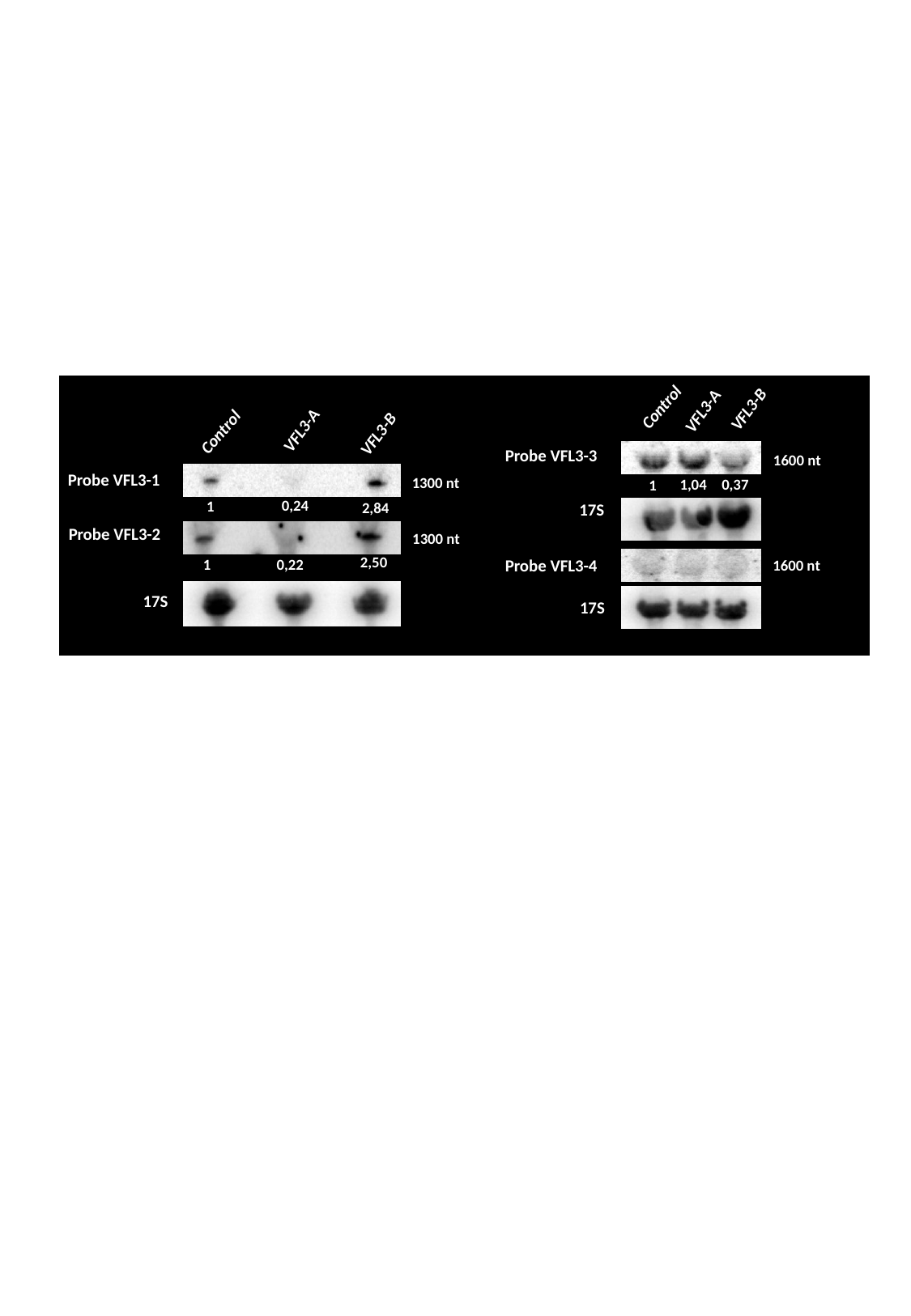

Control
VFL3-B
VFL3-A
VFL3-A
VFL3-B
Control
Probe VFL3-3
1600 nt
Probe VFL3-1
1300 nt
1,04
0,37
1
0,24
1
2,84
17S
Probe VFL3-2
1300 nt
2,50
Probe VFL3-4
1
0,22
1600 nt
17S
17S

Supplement: Supplementary file 6 — Additional file 6: Figure S6. VFL3-A and VFL3-B RNAi efficiencies. Efficiency of the VFL3-A and VFL3-B RNAi vectors to inactivate their target sequences was tested by northern Blots. RNA extracted from cells inactivated for VFL3-A family (VFL3-1 and VFL3-2 genes), VFL3-B family (VFL3-3 and VFL3-4 genes) and ND7 (a gene involved in trichocyst discharge used as control) were transferred on blots and hybridize with 32P-labelled probes. Details for all the probes are in Methods. Hybridization signals were normalized using 17S rRNA. Numbers indicate the rate of target expression in RNAi-treated cells, relative to the control. RNAi triggered either by VFL3-1 or VFL3-2 (VFL3-A family) results in ~75% decrease in the total amount of VFL3-1 and VFL3-2 mRNA but does not reduce VFL3-3 and VFL3-4 (VFL3-B family) mRNA. RNAi triggered VFL3-3 result in a 63% decrease in the total amount of VFL3-3 mRNA but not reduce VFL3-1 and VFL3-2 (VFL3-A family) indicating that the probes are specific of each family. The weak signal observed with the VFL3-4 probe indicates that the VFL3-4 gene is poorly expressed. [file 13630_2017_50_MOESM6_ESM.pptx]

## Slide 1
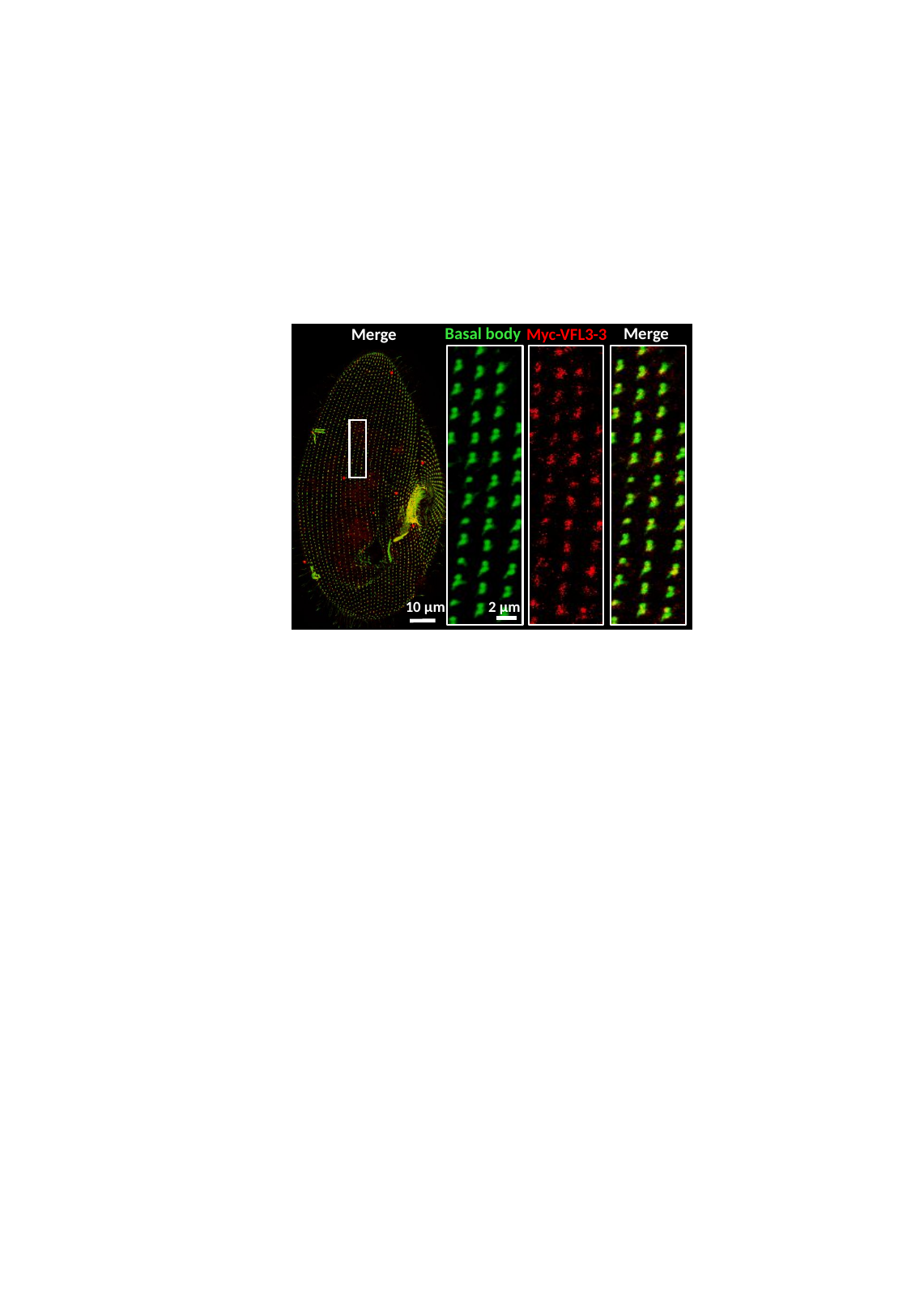

Basal body
Merge
Myc-VFL3-3
2 µm
10 µm
Merge

Supplement: Supplementary file 7 — Additional file 7: Figure S7. Localization of Myc-VFL3-3. Projection of confocal sections through transformants expressing Myc-VFL3-3 fixed and labelles with 1D5 (basal body) and anti-Myc antibody (Myc-VFL3-3). The Myc signal colocalizes with the 1D5 labelling at all basal bodies. [file 13630_2017_50_MOESM7_ESM.pptx]

## Slide 1
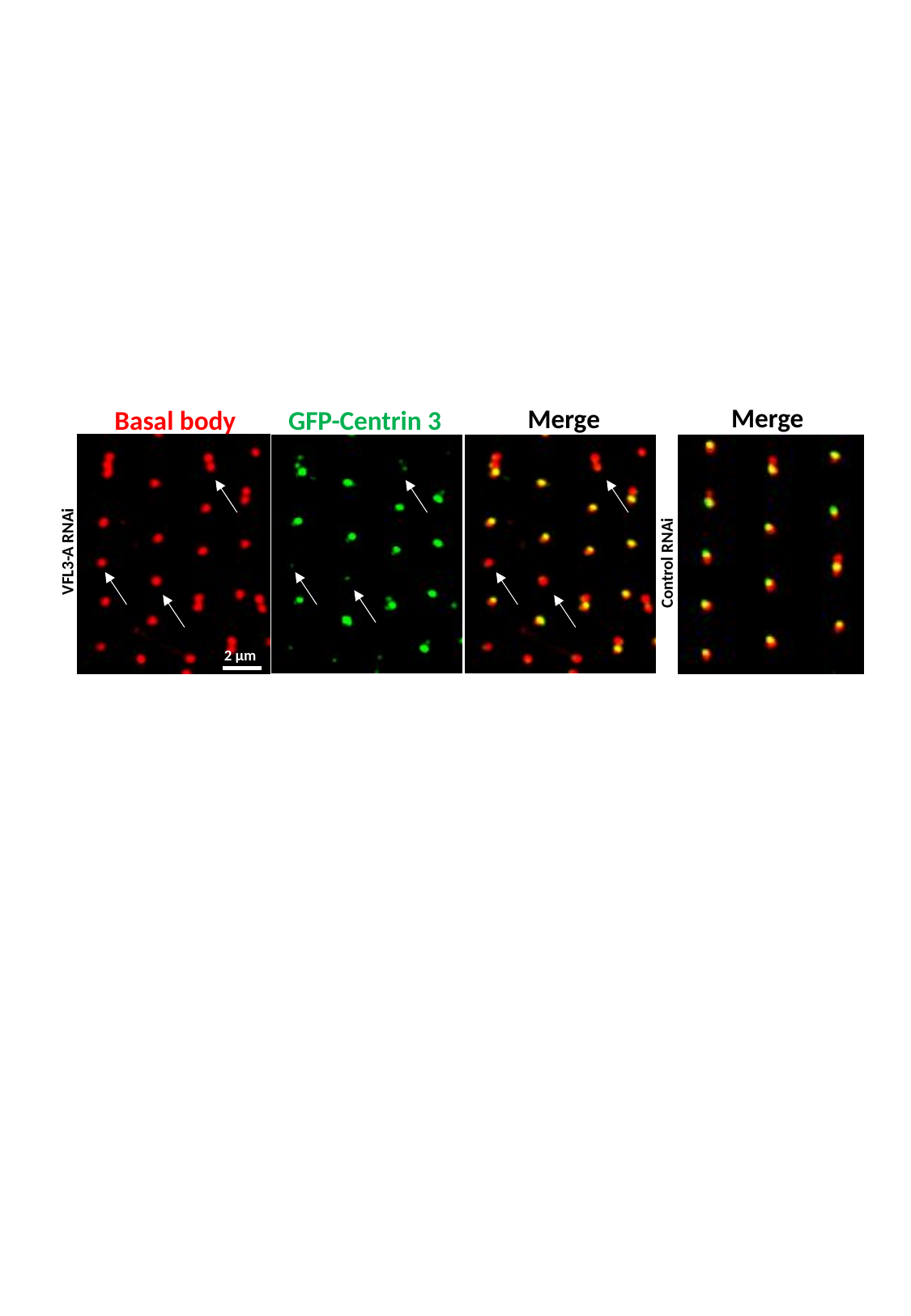

Merge
Merge
Basal body
GFP-Centrin 3
 VFL3-A RNAi
Control RNAi
2 µm

Supplement: Supplementary file 8 — Additional file 8: Figure S8. Relationship between VFL3-A and Centrin 3. Projections of confocal section performed on cells expressing GFP-Centrin3 inactivated by the VFL3 specific vector (left) or by the cpntrol vector (right) on cells labeled by 1D5 (red). In the control cell, parental and newly assembled basal bodies retained the GFP signal. Inactivation of the VFL3-A isoforms in GFP-Centrin 3 expressing cells induces a reduction of the GFP signal in the newly assembled basal bodies (arrows). [file 13630_2017_50_MOESM8_ESM.pptx]
