# Supplementary material for: Basal body positioning and anchoring in the multiciliated cell Paramecium tetraurelia: roles of OFD1 and VFL3
Source: Cilia. 2017 Mar 30;6:6. doi: 10.1186/s13630-017-0050-z (PMC5374602; doi:10.1186/s13630-017-0050-z)
Supplement: Supplementary file 5 — Additional file 5: Figure S5. Relationships of OFD1 with Centrin 2 and FOR20. Images are projection of confocal sections In all experiments, the GFP signal was observed on cells labeled by 1D5 (red). In the control cells all the basal bodies retained the GFP signal. Inactivation of OFD1 in GFP-Centrin2 expressing cells and inactivation of Centrin2 in GFP-OFD1 expressing cells show that the GFP signal is retained in all basal bodies (arrows) after 2 divisions upon inactivation. In contrast, inactivation of FOR20 in GFP-OFD1 expressing cells and inactivation of OFD1 in GFP-FOR20 expressing cells reveal a reduction of the GFP labelling on numerous basal bodies (arrows). As explain in figure S4, the coexistence of basal bodies brightly labelled, as in control cells, and basal bodies harboring a reduced signal is due to the coexistence in the same cell of parental basal bodies and new basal bodies assembled during the inactivation. In inactivated cells, the misorientation and mispositioning of basal bodies with regard to the cell surface explain that the GFP and 1D5 labelling do not strictly overlap since FOR20 and OFD1 are associated with the distal part of the basal bodies. [file 13630_2017_50_MOESM5_ESM.pptx]

## Slide 1
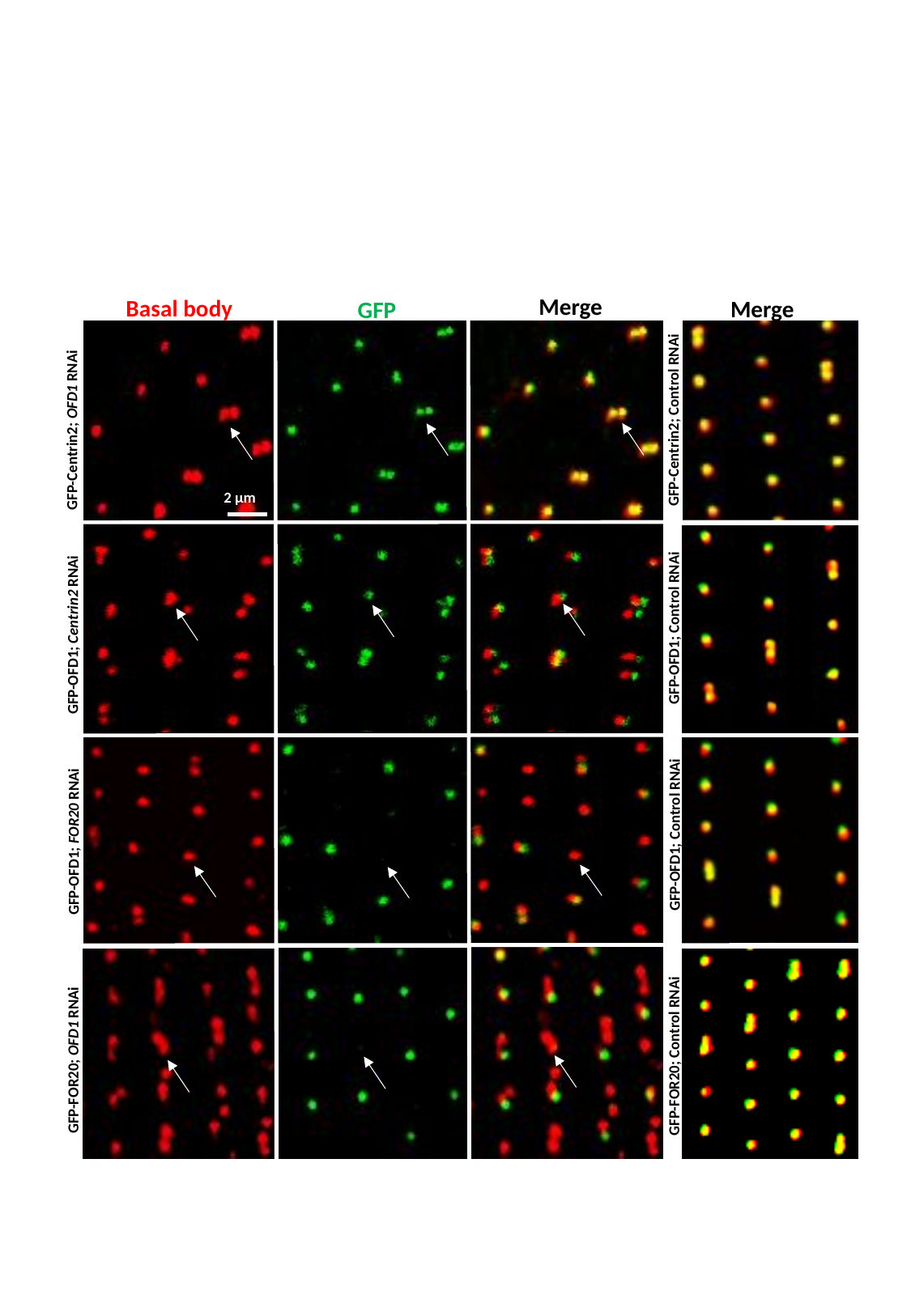

Merge
Basal body
Merge
GFP
GFP-Centrin2; Control RNAi
GFP-Centrin2; OFD1 RNAi
2 µm
GFP-OFD1; Control RNAi
GFP-OFD1; Centrin2 RNAi
GFP-OFD1; Control RNAi
GFP-OFD1; FOR20 RNAi
GFP-FOR20; OFD1 RNAi
GFP-FOR20; Control RNAi
